# Low dimensional representations along intrinsic reaction coordinates and molecular dynamics trajectories using interatomic distance matrices†
†Electronic supplementary information (ESI) available: The ESI contains the following input xyz files and *PathReducer* output xyz files, for mass-weighted and not mass-weighted coordinates and for both “Cartesians” and “Distances” inputs to PCA: (1) input files: (a) malonaldehyde system IRC, (b) S_N_2 system (i) IRC (ii) MD trajectory, (c) N_2_O–acrylonitrile system dihedral scan, (d) cyclopropylidene bifurcation system (i) IRC (ii) four MD trajectories (A–D). (2) Output files: (a) malonaldehyde system (i) PC1, PC2, PC3 (ii) PCall (PC1–3 combined), (b) S_N_2 system (i) PC1, PC2, PC3 for IRC (ii) PCall (PC1–3 combined) for IRC (iii) PC1, PC2, PC3 for MD trajectory (iv) PCall (PC1–3 combined) for MD trajectory, (c) N_2_O–acrylonitrile system (i) PC1, PC2, PC3 using (ii) PCall (PC1–3 combined), (d) cyclopropylidene bifurcation system (i) PC1, PC2, PC3 for IRC (ii) PCall (PC1–3 combined) for IRC (iii) PC1, PC2, PC3 for four MD trajectories (A–D) (iv) PCall (PC1–3 combined) for four MD trajectories (A–D). Relevant plots for all of these systems and all possible input combinations are also included in the ESI. The input file for doing BOMD simulations in *Gaussian 09* is also included. See DOI: 10.1039/c9sc02742d


**DOI:** 10.1039/c9sc02742d

**Published:** 2019-09-18

**Authors:** Stephanie R. Hare, Lars A. Bratholm, David R. Glowacki, Barry K. Carpenter

**Affiliations:** a University of Bristol School of Chemistry , Cantock's Close , Bristol , UK BS8 1TS; b University of Bristol School of Mathematics , University Walk , Bristol , UK BS8 1TW; c University of Bristol School of Computer Science , Merchant Venturers Building, Woodland Road , Bristol , UK BS8 1UB; d Cardiff University School of Chemistry , Main Building, Park Place , Cardiff , UK CF10 3AT . Email: carpenterb1@cardiff.ac.uk

## Abstract

Most chemical transformations (reactions or conformational changes) that are of interest to researchers have many degrees of freedom, usually too many to visualize without reducing the dimensionality of the system to include only the most important atomic motions. In this article, we describe a method of using Principal Component Analysis (PCA) for analyzing a series of molecular geometries (*e.g.*, a reaction pathway or molecular dynamics trajectory) and determining the reduced dimensional space that captures the most structural variance in the fewest dimensions. The software written to carry out this method is called *PathReducer*, which permits (1) visualizing the geometries in a reduced dimensional space, (2) determining the axes that make up the reduced dimensional space, and (3) projecting the series of geometries into the low-dimensional space for visualization. We investigated two options to represent molecular structures within *PathReducer*: aligned Cartesian coordinates and matrices of interatomic distances. We found that interatomic distance matrices better captured non-linear motions in a smaller number of dimensions. To demonstrate the utility of *PathReducer*, we have carried out a number of applications where we have projected molecular dynamics trajectories into a reduced dimensional space defined by an intrinsic reaction coordinate. The visualizations provided by this analysis show that dynamic paths can differ greatly from the minimum energy pathway on a potential energy surface. Viewing intrinsic reaction coordinates and trajectories in this way provides a quick way to gather qualitative information about the pathways trajectories take relative to a minimum energy path. Given that the outputs from PCA are linear combinations of the input molecular structure coordinates (*i.e.*, Cartesian coordinates or interatomic distances), they can be easily transferred to other types of calculations that require the definition of a reduced dimensional space (*e.g.*, biased molecular dynamics simulations).

## Abstract 2

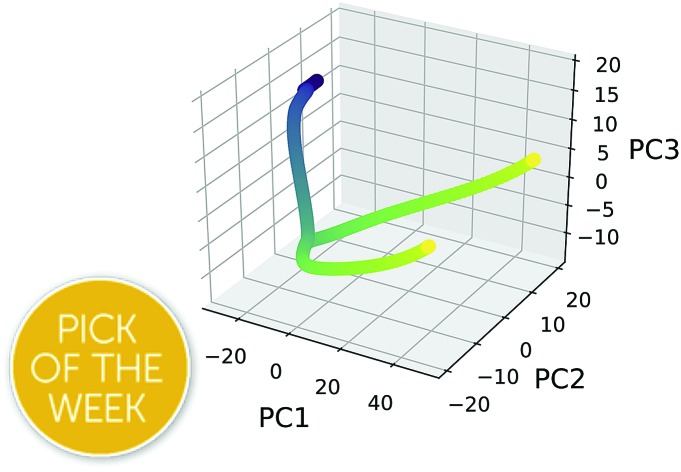
Principal Component Analysis on a series of molecular geometries (*e.g.*, a reaction coordinate or trajectory) provides maximum structural variance in the fewest dimensions, and so can offer an objective, comprehensible depiction of the transformation.

## 1. Introduction

Chemical reaction pathways and structural transformations occurring on hyperdimensional potential energy surfaces (PESs) can be difficult to comprehend due to the high number of degrees of freedom available in most molecular systems. The use of reaction coordinate diagrams and reduced dimensional potential energy surface scans^1^ (RDPESs) has already demonstrated the utility of viewing chemical reactions in a small number of dimensions. These approximate RDPESs are often made by incrementally varying a small number of geometric features and plotting the values of potential energy as a function of these features to generate a low-dimensional surface. For example, a recent paper by Liu *et al.* details a method of using RDPESs on which to conduct *ab initio* molecular dynamics (MD) simulations where the RDPESs were constructed using geometric coordinates “chosen based on the chemical knowledge of the system.”^2^ In addition to generating RDPESs, similar approaches (*e.g.*, choosing specific bond distances, angles, and dihedrals along the course of trajectories as in ref. 3–8) are often used to plot several MD trajectories, and to carry out free energy sampling [*e.g.*, using methods like umbrella sampling,^9^ metadynamics,^10^ boxed molecular dynamics (BXD),^11^,^12^ forward flux sampling,^13^ milestoning,^14^ all of which require a well-defined reduced dimensional space of collective variables from which to sample]. In general, these sorts of analyses tend to rely heavily on user input, *i.e.*, the person making the surface uses their chemical intuition to pick geometric criteria that will make the analysis useful. However, by inferring the geometric changes most important to a reaction and calculating the energy of structures along those coordinates, one runs the risk of confirming one's own biases, and neglecting potentially important degrees of freedom. In a variety of realms, it is therefore useful to have an automated method for generating low-dimensional representations to describe structural changes along molecular pathways that is *quantitatively and a priori* derived from the input data.

In this article, we outline a dimensionality reduction method incorporating principal component analysis (PCA). PCA is an extremely popular method in various fields: in experimental biology, PCA is used to determine the effects of different gene expressions.^15^–^17^ In analytical chemistry, PCA is central in the development of quantitative structure activity relationship (QSAR) models, of particular utility in the pharmaceutical industry.^18^–^21^ Perhaps most closely related to this study is the use of PCA in computational biology, to capture essential motions of a protein in MD simulations.^22^–^24^ There are, however, still some key limitations of PCA: first, it is assumed that the relationships between features of the data points are linear. Second, principal components must be orthogonal to one another, so some types of coupled motions may not be well-described (*i.e.*, related to the first point, motions that are coupled in non-linear relationships). Third, because PCA aims to pick principal components along which the variance of the data is maximized, some shapes of the data distribution can end up being described poorly (*e.g.*, two “bands” of data, or stacked “pancakes” of data points).^25^–^27^ Despite these limitations, for the applications described herein, PCA does an excellent job of defining a reduced dimensional space, without losing too much structural information along the chemical pathways examined, and the issue of capturing non-linear motions can be mitigated by adjusting the representations of molecular structures that are input to PCA. Despite its utility and the fact that reaction coordinates of small-molecule systems are not as susceptible as those of larger systems to suffer from the aforementioned limitations, as far as we know, PCA is not commonly utilized for the visualization of small-molecule chemical change.

For computational studies of large biomolecular systems occurring over long timescales, a suitable choice of collective variables is necessary for modelling dynamics, and thus many dimensionality reduction techniques in addition to PCA have been explored in the field. For example, in the realm of Markov state models, many in the computational community have chosen to employ time-lagged (or time-structure based) independent component analysis (TICA)^28^ rather than PCA. TICA aims to maximize the autocorrelation for a given lag time, rather than the variance, and so is better able to resolve slow timescale events, which is better for capturing the slow dynamics of large molecules like enzymes.^26^,^29^ Diffusion maps constitute a dimensionality reduction technique that does not assume the data points to be related linearly, but instead seeks to determine the manifold in which the data live.^30^–^32^ For the small-molecule applications discussed below, where we are not considering very large systems occurring over large timescales and particularly because we are focusing on intrinsic reaction coordinates (IRCs) rather than MD trajectories to define a reduced dimensional space, we chose to use PCA in order to determine the optimal reduced dimensional space for these example systems. The methods described herein are provided in an open-source software package named *PathReducer*, which allows the user to decide whether their system is best described by linear combinations of Cartesian coordinates or squared interatomic distances, and also whether they would like these inputs to be mass-weighted prior to processing. The merits of all options as applied to several example systems are discussed in the results section, below.

In this paper, we have three principal goals. The first is to introduce the application of PCA into the field of small-molecule computational chemistry, where its value may not have been as widely recognized as it has been in computational biology. The second is to show the utility of using PCA to analyze and characterize chemical pathway data. In particular, we show that a variant of PCA in which the input data are squared internal distances can have advantages over the version in which Cartesian coordinates are used. Additionally, by using a reduced dimensional space defined by an IRC and projecting MD trajectory data into this space, one can quickly classify the routes taken by trajectories compared to the minimum energy path. The third objective is to provide our code, *PathReducer*: an easy-to-use code for computational chemists to reduce the dimensionality of their molecular systems.

## 2. *PathReducer*: dimensionality reduction software

**Fig. 1:**
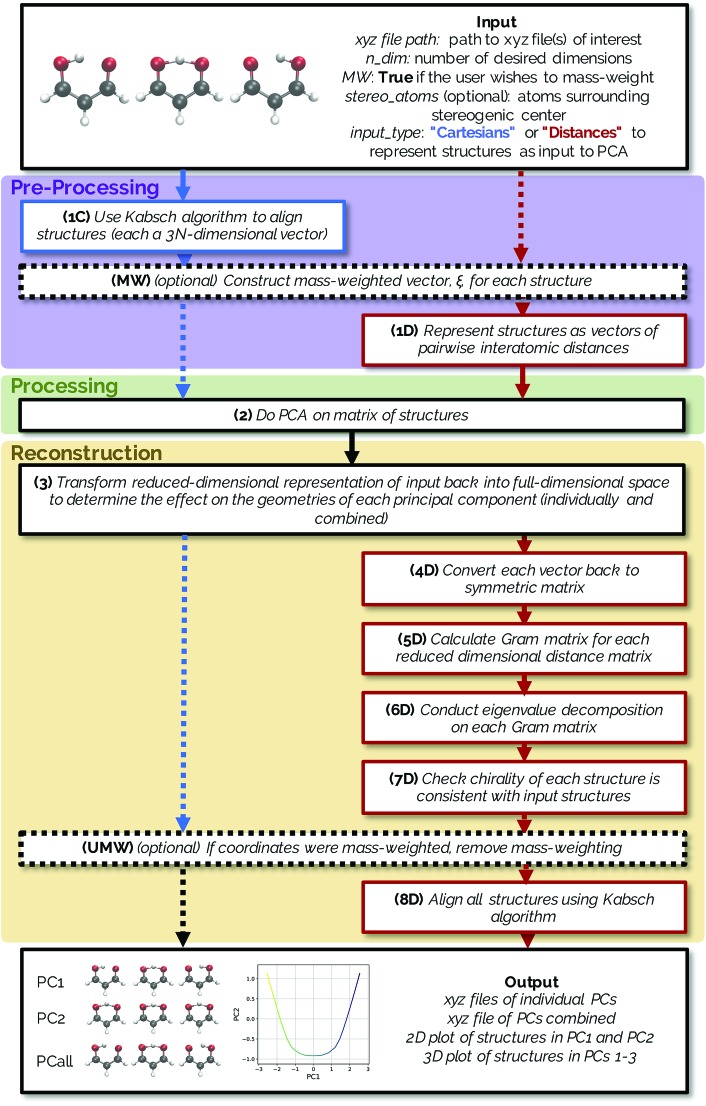
A flowchart indicating how *PathReducer* works. The blue arrows/boxes represent the procedure used if the user specifies a “Cartesians” input to PCA and the red arrows/boxes represent the path taken with a “Distances” input specified. Black arrows/boxes are parts of the method shared by both input types.

The methods described below are freely available in an open source Python package named *PathReducer*, with further details in the ESI.† While there are many dimensionality reduction packages already available in the scikit-learn^33^,^34^ library in Python, the present software is specifically designed to process trajectories of small molecules and generate visualizations thereof. The RMSD Python package, which calculates the RMSD between structures and does alignments using a variety of possible methods, was also utilized in the making of this code for structural alignments using the Kabsch algorithm.^35^ A flowchart illustrating how *PathReducer* works is shown in Fig. 1.

### 2.1 Input

*PathReducer* takes as input the following:

(a) A series of molecular geometries (*e.g.*, an IRC, a trajectory, a relaxed potential energy surface scan) in xyz file format;

(b) *n*_dim_, the number of dimensions for the low-dimensional space (often two or three dimensions would be most useful for visualization);

(c) Whether the user wants PCA analysis to be carried out on mass weighted input coordinates;

(d) Optional labels of four atoms surrounding a stereogenic center of the molecule in order to define chirality (this is only necessary when defining the molecular structures as squared interatomic distance matrices, discussed in more detail below);

(e) The representation of the IRC/trajectory upon which the user wants to perform PCA. The user can specify that PCA be performed on the aligned Cartesian coordinates of the structures (keyword “Cartesians”) or on the upper triangle of the squared interatomic distance matrices of the structures (keyword “Distances”).

The full distance matrix representation is less suitable for very large systems as the size of the representations scales as 
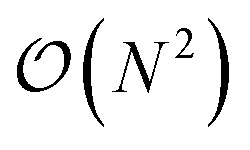
, with *N* being the number atoms, whereas the aligned Cartesian coordinate representation scales with 
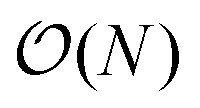
. Using internal distances, however, provides a more accurate reduced dimensional representation in fewer dimensions when non-linear motions (*e.g.* torsions) are involved in the reaction pathway. Additionally, the output from using interatomic distance matrices as input to PCA is more suitable for use in free energy sampling methods since the representation is rotationally and translationally invariant.

### 2.2 Pre-processing

Both methods have the option to mass-weight the Cartesian coordinates prior to processing by PCA, but mass-weighting must occur after structural alignment. If the specified input is “Cartesians”, the Cartesian coordinates of the structures are represented as 3*N*-dimensional vectors and aligned using the Kabsch algorithm (step 1C in Fig. 1).^36^ If the user chooses to mass-weight, the Cartesian coordinates are at this point transformed according to the following equation:1
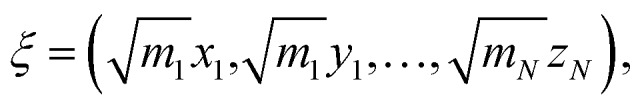
where *ξ* is the 3*N*-dimensional vector containing the mass-weighted coordinates for a single structure along the IRC/trajectory, *m*_*N*_ is the mass of atom *N*, and *N* is the number of atoms in the system (MW step in Fig. 1). If the specified input is “Distances”, rather than using the 3*N*-dimensional aligned Cartesian coordinate vectors to represent each structure along the IRC, each structure is represented as a squared internal distance matrix with each element representing the squared Euclidean distance between an atom pair of the molecule, generating an (*N* × *N*)-dimensional distance matrix for each input structure (step 1D in Fig. 1). Because each interatomic distance matrix is symmetric with its diagonal elements being zero, the upper triangle of each matrix can be flattened to a vector of length 
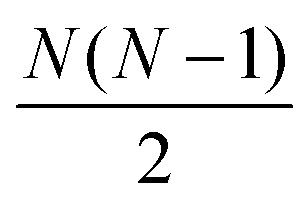
 containing all of the pairwise distances.

### 2.3 Processing

The data processing step (step 2 in Fig. 1) involves performing PCA on the [*n* × 3*N*] or 
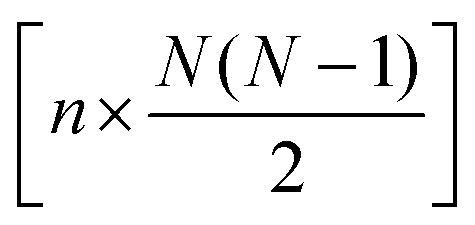
-dimensional matrix of structures, *n* being the number of structures from the input xyz file. Because PCA is well-described in the literature,^25^,^27^ we will only give a brief summary of the method here. PCA takes a set of *n* observations with *p* variables (in our case, *n* structures along an IRC/trajectory with 3*N* Cartesian coordinates or 
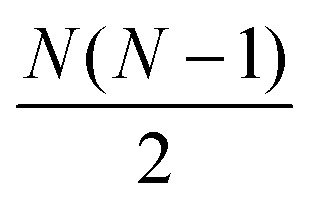
 interatomic distances) and returns an orthogonal basis that maximizes the variance captured by the minimum number of principal components. This transformation is accomplished by a diagonalization of the mean-centered covariance matrix *C* to generate a new orthogonal coordinate system as follows:

2 *Λ*_*C*_ = *U*_*C*_*CU*T*C*,

where *U*_*C*_ is the matrix of eigenvectors, each of which represents a new coordinate that corresponds to a linear combination of the original variables, and *Λ*_*C*_ is the diagonal matrix of the corresponding eigenvalues (*λ*_*C*_) of *C*. In this case, the principal components are linear combinations of Cartesian coordinates or squared interatomic distances. The corresponding eigenvalues correspond to the proportion of the total variance of the system that is captured by each eigenvector. The amount of variance captured by each eigenvector is contained in the eigenvector's corresponding eigenvalue, *λ*_*C*_. What is often referred to as the “goodness of fit” (G.o.F.) or the “variance explained” by the reduced dimensional model corresponds to the sum of the eigenvalues of the number of eigenvectors used in the reduced dimensional space (that is, the fraction of variance captured by the *n*_dim_ principal components chosen):^37^3
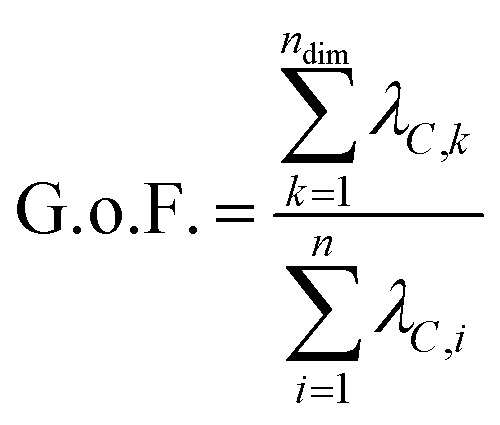



### 2.4 Reconstruction

The reduced-dimensional IRC/trajectory can then be transformed back into the original, full-dimensional space to reconstruct the effect of individual principal components on the molecular geometries using the following expression (step 3 in Fig. 1):

4 *X[combining tilde]* = *T*_*i*_·*W*_*i*_ + *X[combining macron]*,

where *X[combining tilde]* is the [*n* × 3*N*] or 
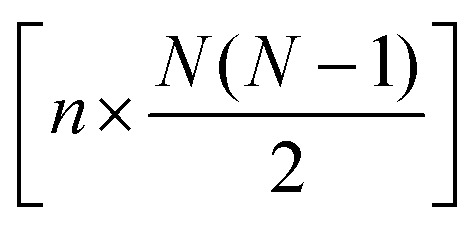
-dimensional matrix of reduced dimensional structures transformed into the original, full-dimensional space, *T*_*i*_ is the [*n* × 1]-dimensional matrix of structures represented by the *i*th principal component, *W*_*i*_ is the [1 × 3*N*] or 
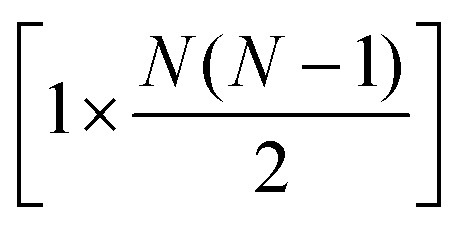
-dimensional matrix corresponding to weights of the *i*th principal component, and *X[combining macron]* is the mean structure of the original dataset. Similarly, the following expression is used to reconstruct the combined effect of the *n*_dim_ principal components:

5 *X[combining tilde]* = *T*_*i*:*n*_dim__·*W*_*i*:*n*_dim__ + *X[combining macron]*,

where *T*_*i*:*n*_dim__ is the [*n* × *n*_dim_]-dimensional matrix of structures represented by all *n*_dim_ principal components and *W*_*i*:*n*_dim__ is the [*n*_dim_ × 3*N*] or 
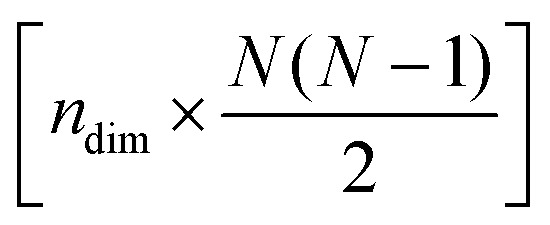
-dimensional matrix containing the weights of the *n*_dim_ principal components.

If using a “Cartesians” input to PCA, this is the last step prior to generating output because the reconstructed structures are in Cartesian space. In the case of using the “Distances” input, the structures that have been transformed into reduced dimensional space at this point are still vectors representing the upper triangle of interatomic distance matrices, and so each row then needs to be converted from squared distances to Cartesian coordinates.^38^ These steps represent the most computationally expensive part of the procedure, as a matrix diagonalization must be done for each molecular structure (step 7D in Fig. 1). The reconstruction of Cartesian coordinates from the flattened, reduced dimensional distance matrices requires the following: each vector is converted back into a square, symmetric matrix with zeroes along the diagonal (step 4D in Fig. 1). The Gram matrix, *G*, for each internal distance matrix is then calculated by:6
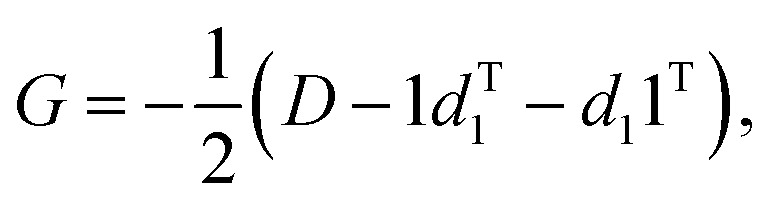
where *D* represents the interatomic distance matrix and *d*_1_ is the first column of *D* (step 5D in Fig. 1). An eigenvalue decomposition (EVD) is then conducted on *G* (step 6D in Fig. 1) as follows:

7 *Λ*_*G*_ = *U*_*G*_*GU*T*G*

The approximate reconstruction of the Cartesian coordinates is given by the first three columns of the matrix generated by taking dot product of the eigenvectors and the square root of their corresponding eigenvalues, *Λ*1/2*GU*T*G*. It should be noted that because the reduced dimensional distance matrix, *D*, is not a *true* distance matrix, but rather what is referred to as a “predistance matrix”,^39^ there will be trailing values in the reconstruction matrix *Λ*1/2*GU*T*G* beyond the first three columns that are a result of the fact that some structural information is lost by reducing the dimensionality of the system. If *D was* a true distance matrix, only the first three columns of *Λ*1/2*GU*T*G* would be nonzero. Additionally, because information about the absolute rotational/reflective configuration is also lost in representing each of the structures as internal distance matrices, these structures will be in an arbitrary rotational/reflective configuration. For the sake of visualization, the Kabsch algorithm,^36^ which determines the optimal rotation matrix to minimize RMSD between pairs of points, is used to align structures along the IRC.

Structures along the reconstructed pathway are reflected if the chirality of the structure at a particular point is not consistent with the analogous structure in the original file (step 7D in Fig. 1). The optional input of four atoms surrounding the stereogenic center are used to determine the chirality of the structure at each point by the method in ref. 40. The sign of the following fourth-grade determinant is used to assign the chirality of the structure:8
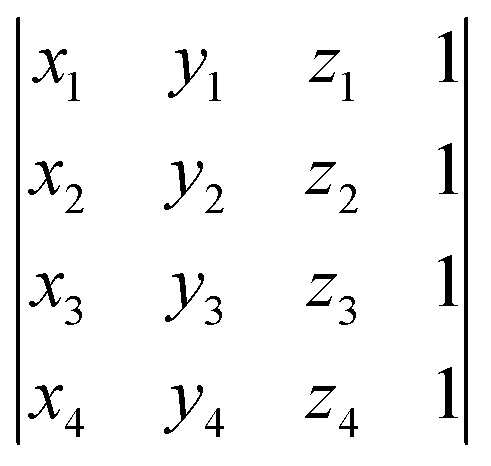
where *x*_*i*_, *y*_*i*_, and *z*_*i*_ represent the Cartesian coordinates of the four atoms surrounding the stereogenic center. This determinant will only be equal to zero when the four atoms used to assign the molecule's chirality are in the same plane.

If coordinates were mass-weighted, mass-weighting of the coordinates is removed according to the following equation (step UMW in Fig. 1):9
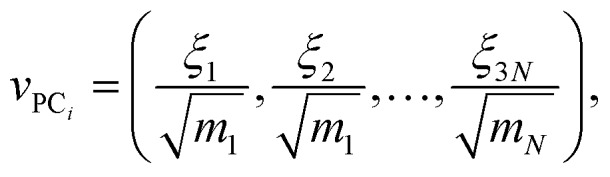
where *v*_PC_*i*__ is the 3*N*-dimensional vector containing the Cartesian coordinates for a structure along the reaction pathway in PC_*i*_, *ξ*_*j*_ is the *j*th component of the 3*N*-dimensional vector containing the mass-weighted coordinates for a single structure along the IRC/trajectory, *m*_*N*_ is the mass of atom *N*, and *N* is the number of atoms in the system. Finally, structures along the reconstructed pathway are aligned using the Kabsch algorithm (step 8D in Fig. 1).

### 2.5 Output

*PathReducer* generates a total of (*n*_dim_ + 1) xyz files from the Cartesian coordinates of the principal components (PCs): the *n*_dim_ PCs individually transformed into the full-dimensional space, as well as the combination of all *n*_dim_ PCs transformed back into the full-dimensional space. These files show the effect of each principal component on the geometries along the trajectory. A plot of the IRC/trajectory in the reduced dimensional space defined by the top two and three PCs is also generated (see below for examples).

## 3. Applications to chemical systems

To illustrate the output of *PathReducer*, we show four examples of systems on which we conduct dimensionality reduction. The first two, “malonaldehyde” and “S_N_2”, are prototypical test systems that have been previously used by Tsutsumi *et al.* to illustrate their dimensionality reduction approach.^41^,^42^ The third is a simple torsional rotation of N_2_O-appended acrylonitrile. The last example is the opening of substituted cyclopropylidene to generate chiral allenes.^43^ The results discussed below utilize coordinates that were *not* mass-weighted. The mass-weighting option is included in case the user wants to define a reduced dimensional space for which the calculated kinetic energy is not dependent on mass. As we were not interested in calculating kinetic energy in our reduced dimensional space, and because some of the systems below include hydrogen movements along the reaction coordinate that we did not want to be dwarfed by the movements of heavy atoms, we chose not to mass-weight the coordinates prior to PCA. Mass-weighting *does* change the results of the dimensionality reduction, as scaling the data on which PCA is conducted changes the reduced dimensional space. In terms of visualization of the pathway in the reduced dimensional space, mass-weighting will give precedence to the movement of heavier atoms; that is, heavier atoms will contribute more to the structural variance along the chemical pathway, which will be reflected in the PCs. For this reason, care should be taken when deciding whether or not it is appropriate to mass-weight the coordinates prior to PCA. Mass-weighting would *not* be appropriate, for example, when hydrogen movements play a large role in the chemical pathway. See the ESI† for mass-weighted results for all of the example systems below.

### 3.1 Quantum mechanical methods for generating IRCs and trajectories

*Gaussian 09* (ref. 44) was used to generate the example IRCs shown below. The malonaldehyde, S_N_2, and cyclopropylidene IRCs were calculated using the MP2 method^45^ with the 6-31+G(d,p) basis set. The MD trajectory for the S_N_2 and cyclopropylidene bifurcation systems were calculated using the Born–Oppenheimer Molecular Dynamics (BOMD) functionality in *Gaussian 09* at the same level of theory as their IRCs. It should be noted that while *ab initio* quantum chemistry methods were used to generate IRCs and MD trajectories in this case, this analysis is not specific to a particular type of calculation or level of theory. All that is needed as input to the method is one or more files containing molecular structures in xyz file format illustrating the transformation(s) of interest.

### 3.2 Malonaldehyde

**Fig. 2:**
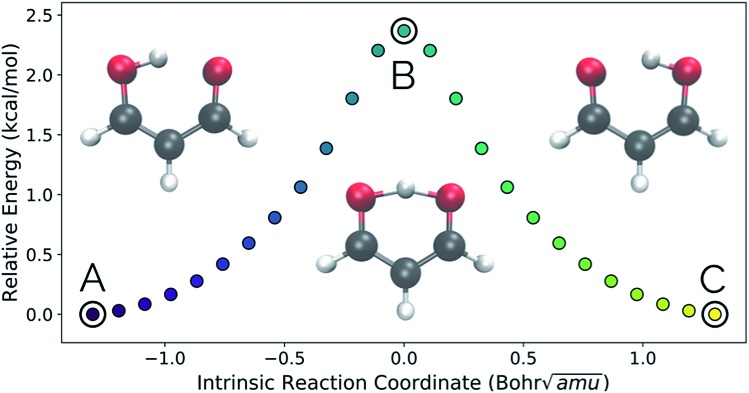
The IRC of the malonaldehyde system. Structures A, B, and C represent reactant, transition state, and product structures, respectively. In this and all similar plots below, purple represents the beginning of an IRC/trajectory and yellow represents the end.

Intramolecular hydrogen transfer between the two oxygens of malonaldehyde is one of the most studied systems in reaction dynamics, owing to the fact that the reaction coordinate is symmetric about the transition state structure, generating indistinguishable molecules. The IRC for this reaction, as well as reactant, transition state, and product structures, can be seen in Fig. 2.

**Fig. 3:**
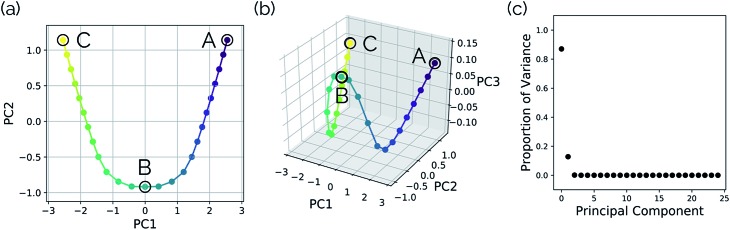
Plots illustrating a projection of the malonaldehyde IRC structures in reduced dimensional space. (a) A two-dimensional plot of the top two and (b) top three principal components of the malonaldehyde system using squared interatomic distances as input to PCA. (c) The proportion of the variance in the IRC data for each individual principal component. In this case, two principal components describe over 99% of the total variance in the data.

Fig. 3a and b show the results obtained when *PathReducer* is used to represent the structures along the malonaldehyde IRC as squared internal distance matrices that are input to PCA. Fig. 3c shows that the first principal component (PC1) describes 87.0% of the variance, while PC2 accounts for 12.8%. As these components capture more than 99% of the total variance in the geometrical changes along the IRC, we conclude that the important molecular motions are captured by this two-dimensional space. Performing PCA on the aligned Cartesian coordinates gives very similar results, which are shown in the ESI.†

**Fig. 4:**
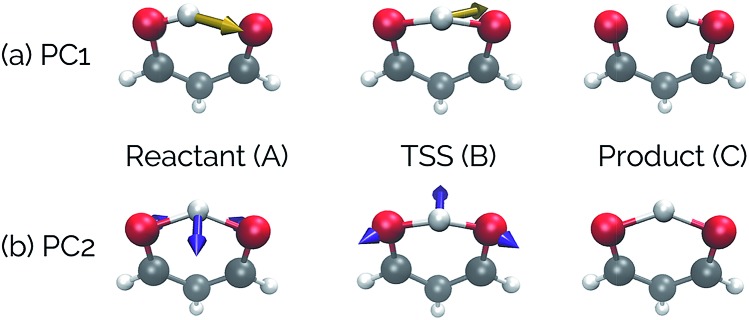
The top two principal components transformed onto the original malonaldehyde IRC. Vectors superimposed on the structures correspond to deformation vectors in going to the next structure along that PC (*e.g.*, the vectors shown on the reactant structure shows the atomic movements necessary to go to the TSS, while the vectors on the TSS are the movements to get to product). Vector magnitudes were adjusted for clarity. These vectors are for illustrative purposes only, as they would change depending on the alignment of the structures along the PC. For videos of these PCs, see ; https://vimeo.com/335614575 for PC1 and ; https://vimeo.com/335614565 for PC2.

Fig. 4 shows that the most significant principal component (PC1) corresponds to motion of the hydrogen atom between the two carbonyl oxygens and alternating single and double bond character of the two C–C bonds. The second most significant principal component (PC2) corresponds predominantly to inward motion of the carbonyl oxygens, where the oxygens are farthest apart in the reactant and product structures and closest together at the transition state structure. For videos of these PCs, see ; https://vimeo.com/335614575 for PC1 and ; https://vimeo.com/335614565 for PC2. See the ESI† for corresponding xyz files of these PCs.

### 3.3 S_N_2 reaction between OH^–^ and CH_3_F

**Fig. 5:**
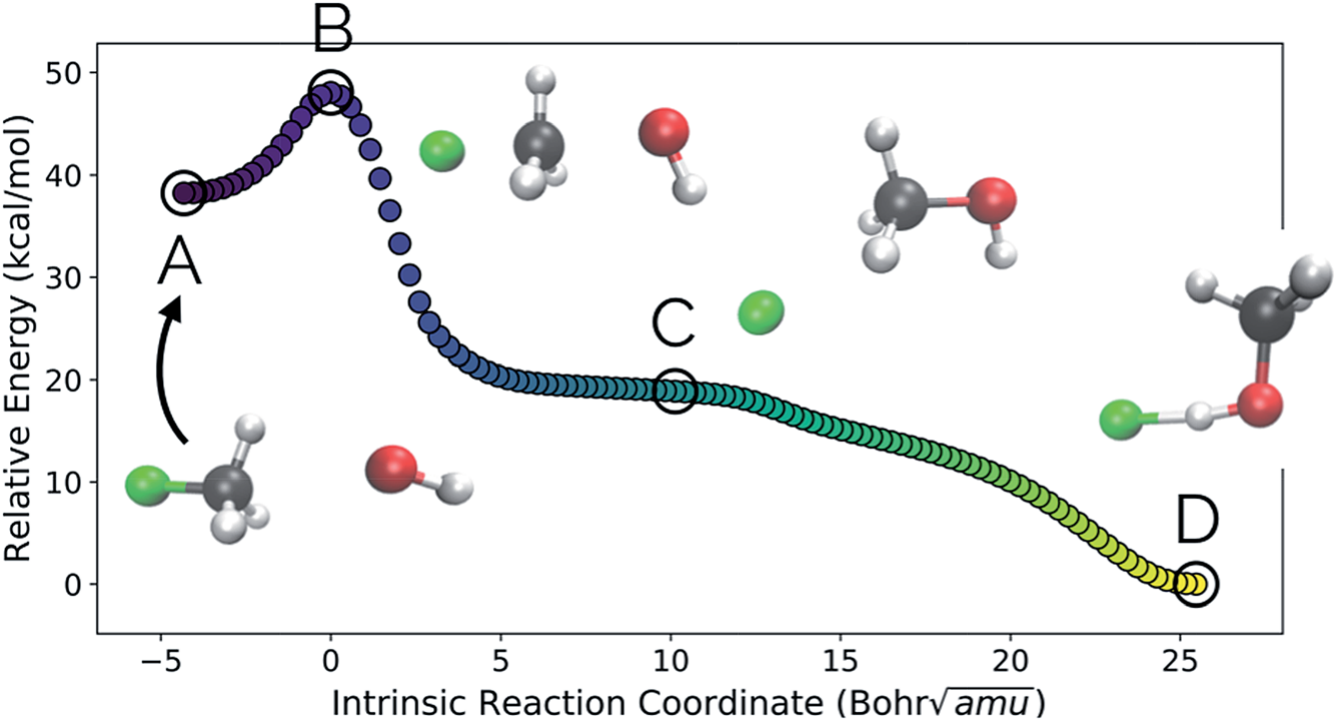
The IRC for the S_N_2 system. Structures A, B, and D represent the reactant, transition state, and product structures (after the fluoride ion orbits the system to hydrogen bond with the hydroxyl group), respectively. Structure C represents the structure where PC2 is at a minimum (Fig. 6), which can be thought of as the structure of the system when the fluoride ion has fully dissociated from the methanol, but has not yet come back to hydrogen bond with the hydroxyl group.

Our second example is the S_N_2 reaction between hydroxide ion and fluoromethane, where hydroxide ion attacks the backside of fluoromethane and releases a fluoride ion (Fig. 5). Modelled in the gas phase, along the IRC, the fluoride ion does not dissociate completely, but rather orbits the newly generated methanol until it finds a suitable location to hydrogen bond with the hydroxyl group. This is not, however, the most common scenario in MD trajectories. Only 10% of MD trajectories conducted by Tsutsumi *et al.* showed the fluoride ion hydrogen bonding with the resultant methanol, while the other 90% had the fluoride dissociating from the system completely.^42^

**Fig. 6:**
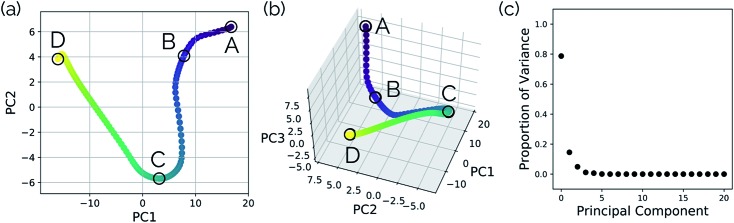
Structures from the S_N_2 IRC shown in Fig. 5 projected into the (a) top two and (b) top three principal components of the S_N_2 system when using squared interatomic distances as the representations of structures that are input to PCA. The locations of structures A, B, C, and D from Fig. 5 with respect to these principal components are labelled. (c) The proportion of variance described by each principal component.

In this system, with squared interatomic distances as input to PCA, PC1 accounted for 78.7% of the variance, PC2 for 14.5%, and PC3 for 4.9% (Fig. 6c, below).

**Fig. 7:**
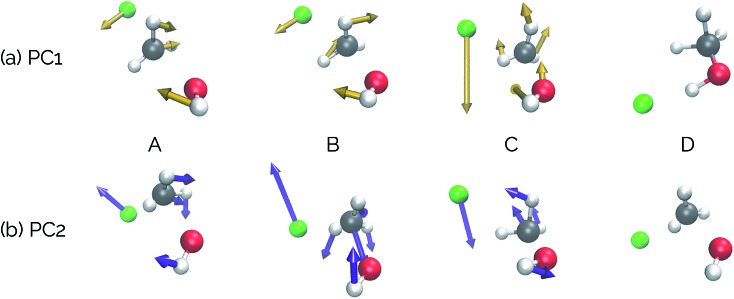
Deformation vectors and geometries of structures A–D represented by PC1 and PC2 along the S_N_2 IRC. The deformation vectors correspond to the atomic motions necessary for the current structure's geometry to form the following structure's geometry (*i.e.*, structure A going to structure B, structure B going to C, *etc.*). Relative vector magnitudes within a frame are quantitative, but are only qualitative between frames (*i.e.*, the magnitude of all vectors in a frame were adjusted by the same factor in order to increase clarity) and should be used as illustrative purposes only, as these vectors are dependent on the final alignment of the structures along the PC. For videos of these PCs, see ; https://vimeo.com/335614633 for PC1 and ; https://vimeo.com/335614625 for PC2.

Visualizations of the geometric changes along the top two principal components can be found in Fig. 7. PC1 represents a pathway that looks quite similar to the original IRC, where the fluoride ion dissociates from methanol and then orbits around the molecule to interact with the hydroxyl group. For a video of PC1, see ; https://vimeo.com/335614633. PC2 represents an almost periodic motion (as can be seen in Fig. 6a, where PC2 starts at a maximum, reaches a minimum, and then returns near to the same maximum) of methyl group pyramidalization and O–H bond stretching. For a video of PC2, see ; https://vimeo.com/335614625. Corresponding xyz files for these PCs can be found in the ESI.†

**Fig. 8:**
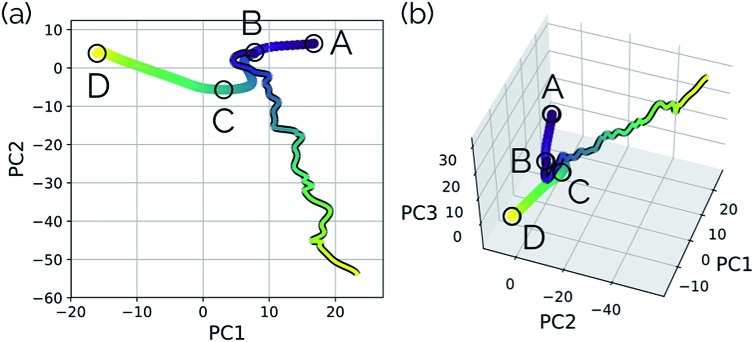
MD trajectory for fluoride dissociation projected into the reduced dimensional space defined by the IRC for the S_N_2 system with respect to (a) the top two principal components and (b) the top three principal components. The IRC, as in previous plots, is shown by the purple to yellow color-mapped line and the trajectory is shown with the same color-mapping, but a black outline. The equivalent plots for PCA on the aligned Cartesian coordinates can be found in the ESI.†

New data for a system can also be projected into a defined reduced dimensional space. To illustrate this, a MD trajectory was initiated from the S_N_2 system's transition state structure (structure B, Fig. 5–7) for 500 steps of 1 fs and propagated in the product direction. As was observed in most of the trajectories calculated by Tsutsumi *et al.*,^42^ after dissociating, the fluoride ion did *not* orbit the resultant methanol and hydrogen bond with the hydroxide group, but rather dissociated completely and did not re-associate for the duration of the trajectory (500 fs). As can be seen in Fig. 8, there is oscillatory movement with the amplitude in the direction of PC1 and almost linear movement in the direction of PC2. This oscillation reflects the excess energy in the forming C–O bond vibration (reflected in PC1) and progression along PC2 is consistent with the C–F distance increasing. Though this reduced dimensional space was defined only by the structures along the S_N_2 IRC, it can be quickly seen from the projection of an MD trajectory in the reduced dimensional space that the dynamical path is very different than the IRC path. In addition to showing that MD trajectory paths can be very different from IRC paths, this example illustrates that *PathReducer* can be used as a straightforward way to classify reaction pathways generated by different types of molecular simulations. Plots of the results when using aligned Cartesian coordinates to represent the molecular structures can be found in the ESI† and look similar to those generated when using squared interatomic distances as input to PCA.

### 3.4 Torsions in the N_2_O–acrylonitrile complex

**Fig. 9:**
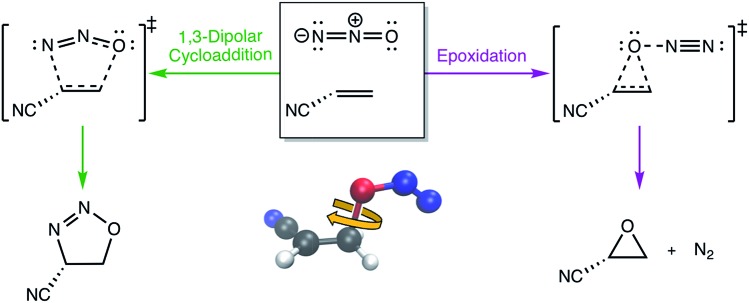
The two possible pathways of N_2_O reacting with acrylonitrile. The ball-and-stick inset illustrates the dihedral angle rotation of the N_2_O–acrylonitrile complex that appears to be the primary geometric coordinate that differentiates the two transition state structures along the competing pathways.

One of the biggest issues that was found in this study with using aligned Cartesian coordinates as input to PCA rather than interatomic distances is how poorly non-linear motions (*e.g.*, torsions) are represented in individual principal components. To illustrate this point, we looked at the dihedral rotation around the C–O bond of a N_2_O–acrylonitrile complex. We chose this system as one that could be interesting to view in reduced dimensions because we posit that this rotation would be a geometric feature that could, in principle, discriminate between two possible reactive pathways: epoxidation or 1,3-dipolar cycloaddition (Fig. 9).

**Fig. 10:**
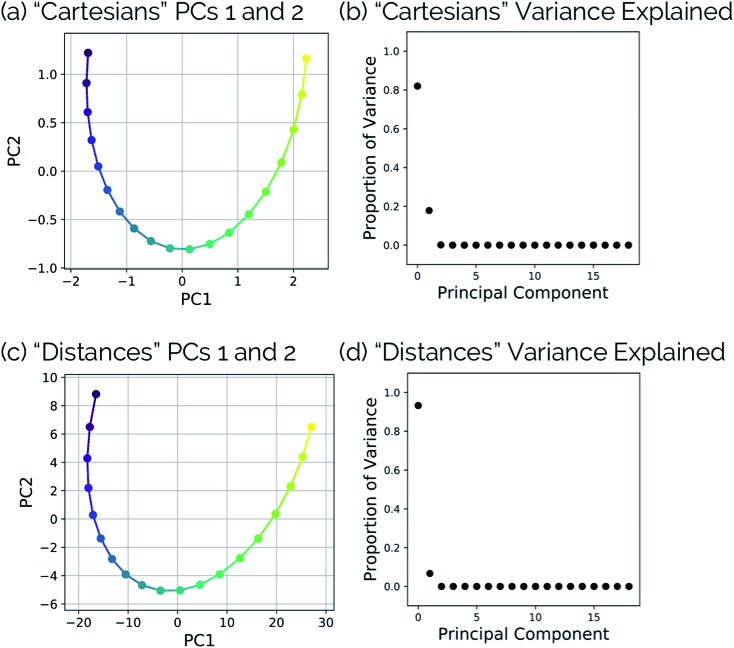
(a) Dihedral scan geometries from Fig. 9 projected into the top two PCs and (b) the proportion of variance explained by each principal component of the N_2_O–acrylonitrile complex system using aligned Cartesian coordinates as input to PCA. (c) Dihedral scan geometries from Fig. 9 projected into the top two PCs and (d) the proportion of variance explained by each principal component of the N_2_O–acrylonitrile complex system using squared interatomic distances as input to PCA.

Fig. 10 shows the IRC projected onto the reduced dimensional space. In this case, two principal components are enough to describe over 99% of the variance in the system. However, using interatomic distances to represent the structures as input to PCA resulted in the first principal component accounting for 93.3% of the variance in the system, whereas an aligned Cartesian coordinates representation of the structures meant the first principal component only accounted for 82.0% of the variance. This result implies that interatomic distance matrices as input to PCA are better for handling torsions in a smaller number of principal components. Thus, if torsions are suspected to be one of the major types of geometric changes along the course of an IRC or trajectory, using the “Distances” input option is likely a better choice (though, if possible, both methods should be screened).

**Fig. 11:**
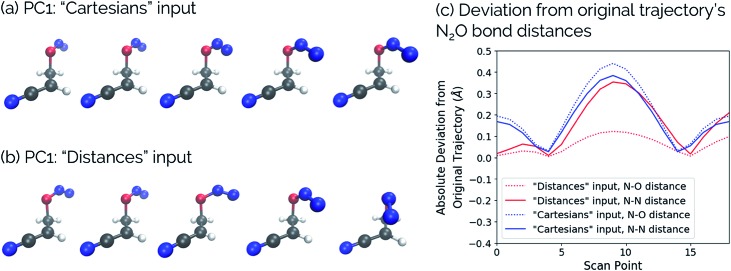
Structural changes along the acrylonitrile IRC transformed into PC1 using (a) aligned Cartesian coordinates and (b) squared interatomic distances as inputs to PCA. (c) The absolute deviation of the N_2_O bond distances in the reconstructed scans compared to the original scan, comparing these bonds using aligned Cartesian coordinates (“Cartesians”) input to PCA (blue) and squared interatomic distances (“Distances”) input (red). For videos of these PCs, see ; https://vimeo.com/336110236 for PC1: “Cartesians” input and ; https://vimeo.com/335614657 for PC1: “Distances” input.

This point can be illustrated by examining the effects of the top principal components on the geometries along the acrylonitrile scan. When performing PCA on the aligned Cartesian coordinates, PC1 significantly compresses the N_2_O moiety during the torsion in order to emulate the effect of a dihedral rotation, while this is not the case when using squared interatomic distances. This is particularly evident in the middle frames shown in Fig. 11a and b. Similarly, a squared interatomic distances representation more accurately preserves the bond distances of the N_2_O moiety (Fig. 11c). See ; https://vimeo.com/336110236 for a video of PC1 using aligned Cartesian coordinates as input to PCA and ; https://vimeo.com/335614657 for PC1 using interatomic distances as input to PCA.

### 3.5 Post-transition state bifurcation in cyclopropylidene ring-opening

**Fig. 12:**
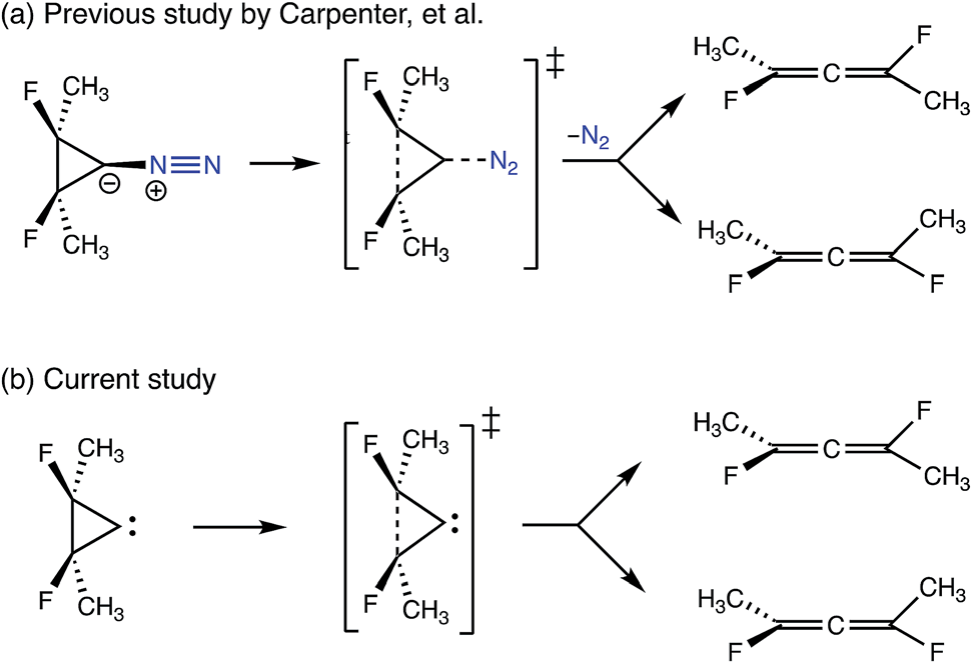
A comparison of the bifurcating reactions in (a) the previous study by Carpenter *et al.*^43^ looking at the effects of chiral solvent on enantiomeric induction and (b) the current study. Both reactions involve the ring-opening of cyclopropylidene to generate enantiomeric allenes, but N_2_ (in blue) was included as a leaving group in the previous study.

The final example to illustrate the utility of this method is a system that exhibits a post-transition state bifurcation.^46^,^47^ This particular system is the ring-opening of cyclopropylidene to generate chiral allenes, which follows up on a reaction previously studied by two of us, investigating the effects of explicit solvent on enantiomeric induction. In the previous study, the concerted, asynchronous transition state structure for the ring-opening event was preceded by N_2_ departure from the carbene carbon, as depicted in Fig. 12.^43^ The system sans N_2_ was chosen to focus on the structural changes along the reaction coordinate of the carbon skeleton (including fluorines).

Systems with post-transition state bifurcations occur in cases where a single transition state structure connects a reactant to two separate products, without any intermediate minima or secondary barriers along the downhill path to either product. If one were to take the upper saddle point structure on the PES as the transition state structure and follow the steepest descent path in the reactant and product directions, where two products are related by symmetry (*e.g.*, enantiomers) the steepest descent path on the product side would pass by a valley-ridge inflection (VRI) point before reaching a minimum. In the case of unsymmetrical bifurcations, there would not be a VRI, but still an additional exit channel with no intervening minima or barriers to overcome. In either case, the IRC would not illustrate the connection between the saddle point and the second possible minimum, as, mathematically, there can only be one steepest descent path. We chose this system to test as input to *PathReducer* because bifurcating reactions represent a class of chemical change whose dynamics are often important, but which have very rarely been visualized using actual structural data and are more often illustrated on qualitative surfaces that illustrate the location of a VRI.^46^,^48^–^54^

**Fig. 13:**
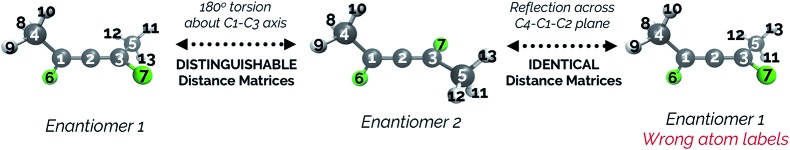
A visual representation of why the structures in the bifurcation IRC following the point where the paths split have distinguishable interatomic distance matrices. In this IRC, enantiomers 1 and 2 are related by a 180° torsion about the *C*_1_–*C*_3_ axis. Though enantiomers 1 and 2 would be considered enantiomers based on atom identities, they are *not* enantiomers when atom *numbering* is taken into account due to the numbering of the atoms on the methyl groups.

While an IRC calculation necessarily picks a single pathway as the minimum energy path, a “bifurcating” IRC could in this case be constructed by a 180° torsion about the *C*_1_–*C*_3_ axis (see Fig. 13) for each structure following the branching point. Note that reflecting each point along the IRC after the point where the paths split would artificially change the atom labels and would cause the distance matrices for the pathway to each product to be identical, and thus would not be able to show the paths splitting. To avoid this, we keep the atom labels consistent with those that would be obtained by a torsional rotation.

**Fig. 14:**
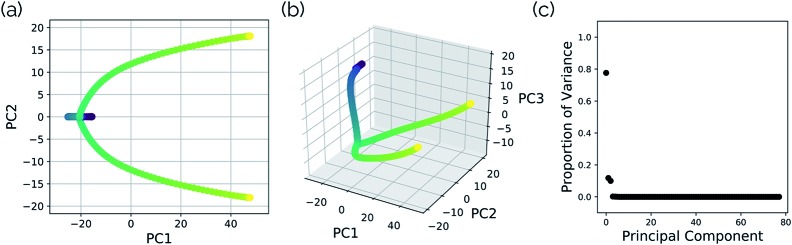
A projection of the structures along the bifurcation IRC into the (a) top two and (b) top three principal components when performing PCA on the IRC structures represented as squared interatomic distances. (c) The proportion of variance described by each principal component in the cyclopropylidene ring-opening bifurcation system.

Fig. 14 illustrates that representing structures along symmetric bifurcating reaction paths using interatomic distance matrices does a good job of illustrating the path “splitting” before leading to the two possible products, whose locations are shown by the yellow ends of the paths. The top three principal components account for 77.6%, 11.8%, and 10.0% of the variance in the IRC, respectively. The equivalent plot using the “Cartesians” input to PCA can be found in the ESI.†

**Fig. 15:**
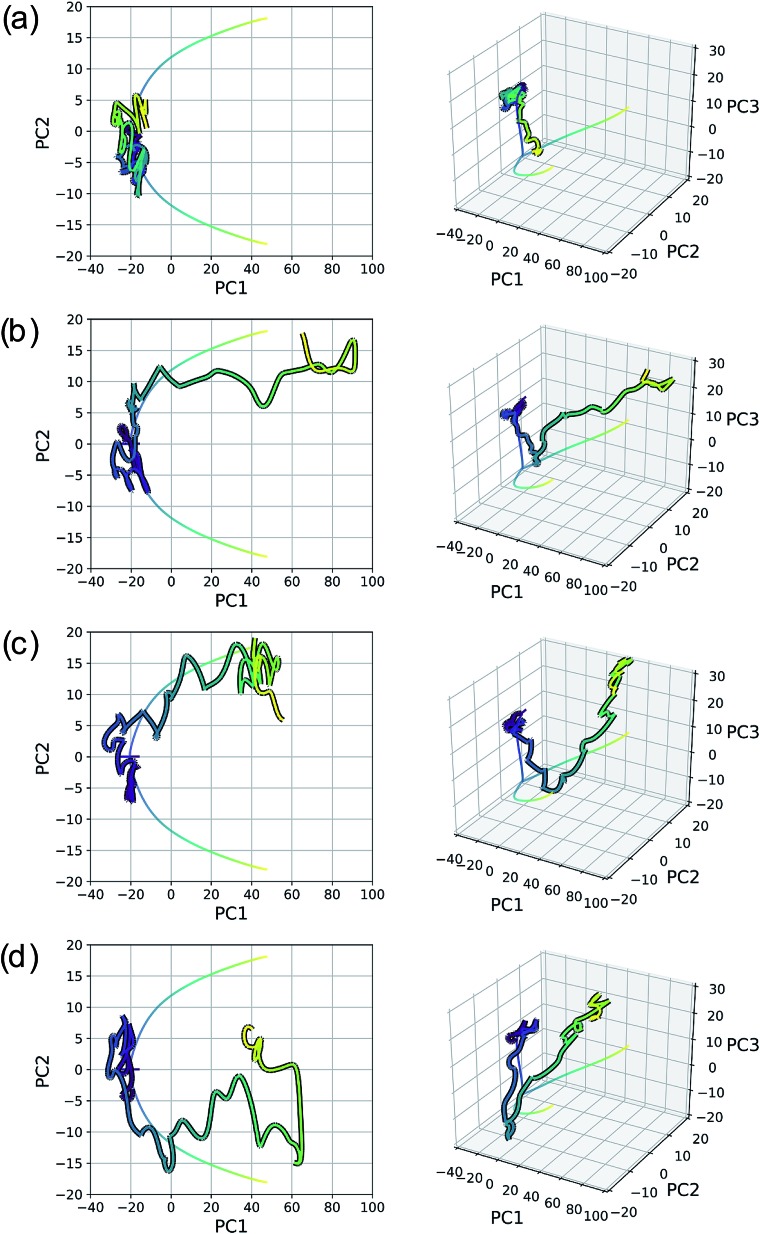
Four 500 fs trajectories projected into the reduced dimensional space defined by the top two (left plots) and three (right plots) PCs of the cyclopropylidene bifurcation IRC, which is represented as a line plot for clarity. Videos of the original MD trajectories being projected here can be found at (a) https://vimeo.com/336131095, (b) ; https://vimeo.com/336131066, (c) ; https://vimeo.com/336131042, and (d) ; https://vimeo.com/336131137.

As with the S_N_2 system, MD trajectories for the cyclopropylidene bifurcation were initiated from the transition state structure and propagated in the product direction. Fig. 15 shows these trajectories projected into the reduced dimensional space defined by the bifurcating IRC. The MD trajectories do not follow the IRC path very closely, indicating that dynamic properties of molecules should not be deduced from IRCs alone. Assigning the product made in each case (if a product is even made) is not entirely straightforward, as illustrated in the original trajectory videos (found at ; https://vimeo.com/336131095 for trajectory A, ; https://vimeo.com/336131066 for trajectory B, ; https://vimeo.com/336131042 for trajectory C, and ; https://vimeo.com/336131137 for trajectory D. See the ESI† for corresponding xyz files). However, projecting these trajectories into the reduced dimensional space defined by the IRC enables rapid qualitative insights into the routes taken by any particular trajectory. Fig. 15a shows a trajectory in which the cyclopropyl ring opens but lingers in the bifurcation region without committing to a clear product pathway. Fig. 15b and c show trajectories which are heading toward generating a single product (enantiomer 2). Fig. 15d is rather different: it goes along the pathway toward enantiomer 1 before traversing the region between the two possible products, a consequence of the fact that the trajectories illustrated in Fig. 15 are run in the gas phase at a constant total energy (NVE ensemble). Therefore, once the molecule goes down the potential energy “hill” after the transition state structure, the molecule has significant excess energy with nowhere to dissipate, which enables interconversion between different product states through high energy geometries.

Seeing MD trajectories projected into a reduced dimensional space defined by an IRC in this way offers a unique perspective on the utility of IRCs compared to MD simulations. While MD trajectories arguably model real, room temperature reactions more accurately by including the effects of finite energy and temperature, this kinetic energy adds noise to the pathway from reactant to product(s). An IRC, however, shows the minimum energy pathway from reactant to product(s); viewed another way, the IRC is the *minimum atomic motion* necessary for a transformation. In this sense, the IRC provides a sort of “skeleton” characterizing the transformation of interest, which is very useful to aid in product classification of MD trajectories. Defining a reduced dimensional space based on an IRC and projecting MD trajectories into this space offers a simple and efficient way to characterize the pathways of MD trajectories in a quantitative comparison to the IRC.

## 4. Conclusions and future work

In conclusion, we have generated a procedure and written software for dimensionality reduction of reaction pathways that is generalizable and can handle specific chemical problems (*e.g.*, torsions and bifurcations). For several examples, we were able to show that this method can reduce the dimensionality of a complex chemical system to a much smaller number of dimensions. For all of the applications outlined herein, two or three dimensions was sufficient to reconstruct the reaction pathway without losing too much information about the structural variance. The principal components generated as a result of this dimensionality reduction method are linear combinations of (potentially mass-weighted) aligned Cartesian coordinates or interatomic distances. For the example systems described, the interatomic distances representation of structures was better than aligned Cartesian coordinates to describe non-linear structural movements, such as torsions. In the future, we plan to use this methodology to choose collective variables to be used in free energy sampling workflows such as metadynamics or boxed molecular dynamics (BXD).^12^ We will also analyze various different types of trajectories [*e.g.*, MD trajectories incorporating explicit solvent, non-adiabatic MD trajectories, gas-surface scattering MD trajectories, user-generated pathways from interactive molecular dynamics in virtual reality (iMD-VR)]. Finally, we would also like to make the code for this method more efficient in order to be better able to analyze enzyme–substrate systems, as similar methods of describing proteins as internal distance matrices have already been utilized.^23^,^55^ Our hope is that *PathReducer* will prove useful for mapping out reaction pathways, as an alternative to relying on chemical intuition to determine geometric changes that are most important along an IRC or trajectory. While improvements are ongoing, we are confident in the broad utility of dimensionality reduction of chemical systems and believe it has the potential to form a useful tool for molecular analysis within the whole of the molecular simulation community.

## Conflicts of interest

There are no conflicts to declare.

## Supplementary Material

Supplementary information
