# Supplementary material for: Low dimensional representations along intrinsic reaction coordinates and molecular dynamics trajectories using interatomic distance matrices
Source: Chem Sci. 2019 Sep 18;10(43):9954–68. doi: 10.1039/c9sc02742d (PMC6991188; doi:10.1039/c9sc02742d)
Supplement: Supplementary file 1 [file SC-010-C9SC02742D-s001.zip › C9SC02742D-SI/PathReducer_SI.docx]

Electronic Supporting Information (ESI)

[Provided xyz files 3](#_Toc9934743)

[Malonaldehyde 3](#_Toc9934744)

[IRC 3](#_Toc9934745)

[“Distances” Input to PathReducer 3](#_Toc9934746)

[Not Mass-Weighted 3](#_Toc9934747)

[Mass-Weighted 3](#_Toc9934748)

[“Cartesians” Input to PathReducer 3](#_Toc9934749)

[Not Mass-Weighted 3](#_Toc9934750)

[Mass-Weighted 4](#_Toc9934751)

[S_N_2 4](#_Toc9934752)

[IRC 4](#_Toc9934753)

[MD Trajectory 4](#_Toc9934754)

[“Distances” Input to PathReducer 4](#_Toc9934755)

[Not Mass-Weighted 4](#_Toc9934756)

[Mass-Weighted 4](#_Toc9934757)

[“Cartesians” Input to PathReducer 5](#_Toc9934758)

[Not Mass-Weighted 5](#_Toc9934759)

[Mass-Weighted 5](#_Toc9934760)

[N_2_O-Appended Acrylonitrile 6](#_Toc9934761)

[Scan 6](#_Toc9934762)

[“Distances” Input to PathReducer 6](#_Toc9934763)

[Not Mass-Weighted 6](#_Toc9934764)

[Mass-Weighted 6](#_Toc9934765)

[“Cartesians” Input to PathReducer 6](#_Toc9934766)

[Not Mass-Weighted 6](#_Toc9934767)

[Mass-Weighted 6](#_Toc9934768)

[Cyclopropylidene Bifurcation 7](#_Toc9934769)

[IRC 7](#_Toc9934770)

[MD trajectories A-D 7](#_Toc9934771)

[“Distances” Input to PathReducer 7](#_Toc9934772)

[Not Mass-Weighted 7](#_Toc9934773)

[Mass-Weighted 7](#_Toc9934774)

[“Cartesians” Input to PathReducer 8](#_Toc9934775)

[Not Mass-Weighted 8](#_Toc9934776)

[Mass-Weighted 9](#_Toc9934777)

[Plots of *PathReducer* Results 10](#_Toc9934778)

[“Distances” Input to PathReducer 10](#_Toc9934779)

[Not Mass-Weighted 10](#_Toc9934780)

[Mass-Weighted 11](#_Toc9934781)

[“Cartesians” Input to PathReducer 11](#_Toc9934782)

[Not Mass-Weighted 11](#_Toc9934783)

[Mass-Weighted 12](#_Toc9934784)

[S_N_2 13](#_Toc9934785)

[“Distances” Input to PathReducer 13](#_Toc9934786)

[Not Mass-Weighted 13](#_Toc9934787)

[Mass-Weighted 14](#_Toc9934788)

[“Cartesians” Input to PathReducer 15](#_Toc9934789)

[Not Mass-Weighted 15](#_Toc9934790)

[Mass-Weighted 16](#_Toc9934791)

[N_2_O-Appended Acrylonitrile 18](#_Toc9934792)

[“Distances” Input to PathReducer 18](#_Toc9934793)

[Not Mass-Weighted 18](#_Toc9934794)

[Mass-Weighted 19](#_Toc9934795)

[“Cartesians” Input to PathReducer 19](#_Toc9934796)

[Not Mass-Weighted 19](#_Toc9934797)

[Mass-Weighted 20](#_Toc9934798)

[Cyclopropylidene Bifurcation 21](#_Toc9934799)

[“Distances” Input to PathReducer 21](#_Toc9934800)

[Not Mass-Weighted 21](#_Toc9934801)

[Mass-Weighted 24](#_Toc9934802)

[“Cartesians” Input to PathReducer 26](#_Toc9934803)

[Not Mass-Weighted 26](#_Toc9934804)

[Mass-Weighted 29](#_Toc9934805)

[Gaussian BOMD input 31](#_Toc9934806)

# Provided xyz files

The following *PathReducer* input and output files are provided:

## Malonaldehyde

### IRC

input_files/malonaldehyde/malonaldehyde_IRC.xyz

### “Distances” Input to PathReducer

#### Not Mass-Weighted

##### PC1

malonaldehyde_IRC_Distances_noMW_output/malonaldehyde_IRC_Distances_noMW_PC1.xyz

##### PC2

malonaldehyde_IRC_Distances_noMW_output/malonaldehyde_IRC_Distances_noMW_PC2.xyz

##### PC3

malonaldehyde_IRC_Distances_noMW_output/malonaldehyde_IRC_Distances_noMW_PC3.xyz

##### PCs 1-3 Combined

malonaldehyde_IRC_Distances_noMW_output/malonaldehyde_IRC_Distances_noMW_all_PCs.xyz

#### Mass-Weighted

##### PC1

malonaldehyde_IRC_Distances_MW_output/malonaldehyde_IRC_Distances_MW_PC1.xyz

##### PC2

malonaldehyde_IRC_Distances_MW_output/malonaldehyde_IRC_Distances_MW_PC2.xyz

##### PC3

malonaldehyde_IRC_Distances_MW_output/malonaldehyde_IRC_Distances_MW_PC3.xyz

##### PCs 1-3 Combined

malonaldehyde_IRC_Distances_MW_output/malonaldehyde_IRC_Distances_MW_all_PCs.xyz

### “Cartesians” Input to PathReducer

#### Not Mass-Weighted

##### PC1

malonaldehyde_IRC_Cartesians_noMW_output/malonaldehyde_IRC_Cartesians_noMW_PC1.xyz

##### PC2

malonaldehyde_IRC_Cartesians_noMW_output/malonaldehyde_IRC_Cartesians_noMW_PC2.xyz

##### PC3

malonaldehyde_IRC_Cartesians_noMW_output/malonaldehyde_IRC_Cartesians_noMW_PC3.xyz

##### PCs 1-3 Combined

malonaldehyde_IRC_Cartesians_noMW_output/malonaldehyde_IRC_Cartesians_noMW_all_PCs.xyz

#### Mass-Weighted

##### PC1

malonaldehyde_IRC_Cartesians_MW_output/malonaldehyde_IRC_Cartesians_MW_PC1.xyz

##### PC2

malonaldehyde_IRC_Cartesians_MW_output/malonaldehyde_IRC_Cartesians_MW_PC2.xyz

##### PC3

malonaldehyde_IRC_Cartesians_MW_output/malonaldehyde_IRC_Cartesians_MW_PC3.xyz

##### PCs 1-3 Combined

malonaldehyde_IRC_Cartesians_MW_output/malonaldehyde_IRC_Cartesians_MW_all_PCs.xyz

## S_N_2

### IRC

input_files/SN2/SN2_IRC.xyz

### MD Trajectory

input_files/SN2/SN2_traj.xyz

### “Distances” Input to PathReducer

#### Not Mass-Weighted

##### PC1

SN2_IRC_Distances_noMW_output/SN2_IRC_Distances_noMW_PC1.xyz

##### PC2

SN2_IRC_Distances_noMW_output/SN2_IRC_Distances_noMW_PC2.xyz

##### PC3

SN2_IRC_Distances_noMW_output/SN2_IRC_Distances_noMW_PC3.xyz

##### PCs 1-3 Combined

SN2_IRC_Distances_noMW_output/SN2_IRC_Distances_noMW_all_PCs.xyz

##### MD Trajectory in PC1

SN2_IRC_Distances_noMW_output/new_data/SN2_traj_Distances_PC1.xyz

##### MD Trajectory in PC2

SN2_IRC_Distances_noMW_output/new_data/SN2_traj_Distances_PC2.xyz

##### MD Trajectory in PC3

SN2_IRC_Distances_noMW_output/new_data/SN2_traj_Distances_PC3.xyz

##### MD Trajectory in PCs 1-3 Combined

SN2_IRC_Distances_noMW_output/new_data/SN2_traj_Distances_all_PCs.xyz

#### Mass-Weighted

##### PC1

SN2_IRC_Distances_MW_output/SN2_IRC_Distances_MW_PC1.xyz

##### PC2

SN2_IRC_Distances_MW_output/SN2_IRC_Distances_MW_PC2.xyz

##### PC3

SN2_IRC_Distances_MW_output/SN2_IRC_Distances_MW_PC3.xyz

##### PCs 1-3 Combined

SN2_IRC_Distances_MW_output/SN2_IRC_Distances_MW_all_PCs.xyz

##### MD Trajectory in PC1

SN2_IRC_Distances_MW_output/new_data/SN2_traj_Distances_PC1.xyz

##### MD Trajectory in PC2

SN2_IRC_Distances_MW_output/new_data/SN2_traj_Distances_PC2.xyz

##### MD Trajectory in PC3

SN2_IRC_Distances_MW_output/new_data/SN2_traj_Distances_PC3.xyz

##### MD Trajectory in PCs 1-3 Combined

SN2_IRC_Distances_MW_output/new_data/SN2_traj_Distances_all_PCs.xyz

### “Cartesians” Input to PathReducer

#### Not Mass-Weighted

##### PC1

SN2_IRC_Cartesians_noMW_output/SN2_IRC_Cartesians_noMW_PC1.xyz

##### PC2

SN2_IRC_Cartesians_noMW_output/SN2_IRC_Cartesians_noMW_PC2.xyz

##### PC3

SN2_IRC_Cartesians_noMW_output/SN2_IRC_Cartesians_noMW_PC3.xyz

##### PCs 1-3 Combined

SN2_IRC_Cartesians_noMW_output/SN2_IRC_Cartesians_noMW_all_PCs.xyz

##### MD Trajectory in PC1

SN2_IRC_Cartesians_noMW_output/new_data/SN2_traj_Cartesians_PC1.xyz

##### MD Trajectory in PC2

SN2_IRC_Cartesians_noMW_output/new_data/SN2_traj_Cartesians_PC2.xyz

##### MD Trajectory in PC3

SN2_IRC_Cartesians_noMW_output/new_data/SN2_traj_Cartesians_PC3.xyz

##### MD Trajectory in PCs 1-3 Combined

SN2_IRC_Cartesians_noMW_output/new_data/SN2_traj_Cartesians_all_PCs.xyz

#### Mass-Weighted

##### PC1

SN2_IRC_Cartesians_MW_output/SN2_IRC_Cartesians_MW_PC1.xyz

##### PC2

SN2_IRC_Cartesians_MW_output/SN2_IRC_Cartesians_MW_PC2.xyz

##### PC3

SN2_IRC_Cartesians_MW_output/SN2_IRC_Cartesians_MW_PC3.xyz

##### PCs 1-3 Combined

SN2_IRC_Cartesians_MW_output/SN2_IRC_Cartesians_MW_all_PCs.xyz

##### MD Trajectory in PC1

SN2_IRC_Cartesians_MW_output/new_data/SN2_traj_Cartesians_PC1.xyz

##### MD Trajectory in PC2

SN2_IRC_Cartesians_MW_output/new_data/SN2_traj_Cartesians_PC2.xyz

##### MD Trajectory in PC3

SN2_IRC_Cartesians_MW_output/new_data/SN2_traj_Cartesians_PC3.xyz

##### MD Trajectory in PCs 1-3 Combined

SN2_IRC_Cartesians_MW_output/new_data/SN2_traj_Cartesians_all_PCs.xyz

## N_2_O-Appended Acrylonitrile

### Scan

input_files/acrylonitrile/acrylonitrile_scan.xyz

### “Distances” Input to PathReducer

#### Not Mass-Weighted

##### PC1

acrylonitrile_scan_Distances_noMW_output/acrylonitrile_scan_Distances_noMW_PC1.xyz

PC2

acrylonitrile_scan_Distances_noMW_output/acrylonitrile_scan_Distances_noMW_PC2.xyz

PC3

acrylonitrile_scan_Distances_noMW_output/acrylonitrile_scan_Distances_noMW_PC3.xyz

##### PCs 1-3 Combined

acrylonitrile_scan_Distances_noMW_output/acrylonitrile_scan_Distances_noMW_all_PCs.xyz

#### Mass-Weighted

##### PC1

acrylonitrile_scan_Distances_MW_output/acrylonitrile_scan_Distances_MW_PC1.xyz

##### PC2

acrylonitrile_scan_Distances_MW_output/acrylonitrile_scan_Distances_MW_PC2.xyz

##### PC3

acrylonitrile_scan_Distances_MW_output/acrylonitrile_scan_Distances_MW_PC3.xyz

##### PCs 1-3 Combined

acrylonitrile_scan_Distances_MW_output/acrylonitrile_scan_Distances_MW_all_PCs.xyz

### “Cartesians” Input to PathReducer

#### Not Mass-Weighted

##### PC1

acrylonitrile_scan_Cartesians_noMW_output/acrylonitrile_scan_Cartesians_noMW_PC1.xyz

##### PC2

acrylonitrile_scan_Cartesians_noMW_output/acrylonitrile_scan_Cartesians_noMW_PC2.xyz

##### PC3

acrylonitrile_scan_Cartesians_noMW_output/acrylonitrile_scan_Cartesians_noMW_PC3.xyz

##### PCs 1-3 Combined

acrylonitrile_scan_Cartesians_noMW_output/acrylonitrile_scan_Cartesians_noMW_all_PCs.xyz

#### Mass-Weighted

##### PC1

acrylonitrile_scan_Cartesians_MW_output/acrylonitrile_scan_Cartesians_MW_PC1.xyz

##### PC2

acrylonitrile_scan_Cartesians_MW_output/acrylonitrile_scan_Cartesians_MW_PC2.xyz

##### PC3

acrylonitrile_scan_Cartesians_MW_output/acrylonitrile_scan_Cartesians_MW_PC3.xyz

##### PCs 1-3 Combined

acrylonitrile_scan_Cartesians_MW_output/acrylonitrile_scan_Cartesians_MW_all_PCs.xyz

## Cyclopropylidene Bifurcation

### IRC

input_files/bifurcation/bifur_IRC.xyz

### MD trajectories A-D

input_files/bifurcation/bifur_traj_A.xyz

input_files/bifurcation/bifur_traj_B.xyz

input_files/bifurcation/bifur_traj_C.xyz

input_files/bifurcation/bifur_traj_D.xyz

### “Distances” Input to PathReducer

#### Not Mass-Weighted

##### PC1

bifur_IRC_Distances_noMW_output/bifur_IRC_Distances_noMW_PC1.xyz

##### PC2

bifur_IRC_Distances_noMW_output/bifur_IRC_Distances_noMW_PC2.xyz

##### PC3

bifur_IRC_Distances_noMW_output/bifur_IRC_Distances_noMW_PC3.xyz

##### PCs 1-3 Combined

bifur_IRC_Distances_noMW_output/bifur_IRC_Distances_noMW_all_PCs.xyz

##### MD Trajectories A-D in PC1-3

bifur_IRC_Distances_noMW_output/new_data/bifur_traj_A_Distances_noMW_PC1.xyz

bifur_IRC_Distances_noMW_output/new_data/bifur_traj_A_Distances_noMW_PC2.xyz

bifur_IRC_Distances_noMW_output/new_data/bifur_traj_A_Distances_noMW_PC3.xyz

bifur_IRC_Distances_noMW_output/new_data/bifur_traj_A_Distances_noMW_all_PCs.xyz

bifur_IRC_Distances_noMW_output/new_data/bifur_traj_B_Distances_noMW_PC1.xyz

bifur_IRC_Distances_noMW_output/new_data/bifur_traj_B_Distances_noMW_PC2.xyz

bifur_IRC_Distances_noMW_output/new_data/bifur_traj_B_Distances_noMW_PC3.xyz

bifur_IRC_Distances_noMW_output/new_data/bifur_traj_B_Distances_noMW_all_PCs.xyz

bifur_IRC_Distances_noMW_output/new_data/bifur_traj_C_Distances_noMW_PC1.xyz

bifur_IRC_Distances_noMW_output/new_data/bifur_traj_C_Distances_noMW_PC2.xyz

bifur_IRC_Distances_noMW_output/new_data/bifur_traj_C_Distances_noMW_PC3.xyz

bifur_IRC_Distances_noMW_output/new_data/bifur_traj_C_Distances_noMW_all_PCs.xyz

bifur_IRC_Distances_noMW_output/new_data/bifur_traj_D_Distances_noMW_PC1.xyz

bifur_IRC_Distances_noMW_output/new_data/bifur_traj_D_Distances_noMW_PC2.xyz

bifur_IRC_Distances_noMW_output/new_data/bifur_traj_D_Distances_noMW_PC3.xyz

bifur_IRC_Distances_noMW_output/new_data/bifur_traj_D_Distances_noMW_all_PCs.xyz

#### Mass-Weighted

##### PC1

bifur_IRC_Distances_MW_output/bifur_IRC_Distances_MW_PC1.xyz

##### PC2

bifur_IRC_Distances_MW_output/bifur_IRC_Distances_MW_PC2.xyz

##### PC3

bifur_IRC_Distances_MW_output/bifur_IRC_Distances_MW_PC3.xyz

##### PCs 1-3 Combined

bifur_IRC_Distances_MW_output/bifur_IRC_Distances_MW_all_PCs.xyz

##### MD Trajectories A-D in PC1-3

bifur_IRC_Distances_MW_output/new_data/bifur_traj_A_Distances_MW_PC1.xyz

bifur_IRC_Distances_MW_output/new_data/bifur_traj_A_Distances_MW_PC2.xyz

bifur_IRC_Distances_MW_output/new_data/bifur_traj_A_Distances_MW_PC3.xyz

bifur_IRC_Distances_MW_output/new_data/bifur_traj_A_Distances_MW_all_PCs.xyz

bifur_IRC_Distances_MW_output/new_data/bifur_traj_B_Distances_MW_PC1.xyz

bifur_IRC_Distances_MW_output/new_data/bifur_traj_B_Distances_MW_PC2.xyz

bifur_IRC_Distances_MW_output/new_data/bifur_traj_B_Distances_MW_PC3.xyz

bifur_IRC_Distances_MW_output/new_data/bifur_traj_B_Distances_MW_all_PCs.xyz

bifur_IRC_Distances_MW_output/new_data/bifur_traj_C_Distances_MW_PC1.xyz

bifur_IRC_Distances_MW_output/new_data/bifur_traj_C_Distances_MW_PC2.xyz

bifur_IRC_Distances_MW_output/new_data/bifur_traj_C_Distances_MW_PC3.xyz

bifur_IRC_Distances_MW_output/new_data/bifur_traj_C_Distances_MW_all_PCs.xyz

bifur_IRC_Distances_MW_output/new_data/bifur_traj_D_Distances_MW_PC1.xyz

bifur_IRC_Distances_MW_output/new_data/bifur_traj_D_Distances_MW_PC2.xyz

bifur_IRC_Distances_MW_output/new_data/bifur_traj_D_Distances_MW_PC3.xyz

bifur_IRC_Distances_MW_output/new_data/bifur_traj_D_Distances_MW_all_PCs.xyz

### “Cartesians” Input to PathReducer

#### Not Mass-Weighted

##### PC1

bifur_IRC_Cartesians_noMW_output/bifur_IRC_Cartesians_noMW_PC1.xyz

##### PC2

bifur_IRC_Cartesians_noMW_output/bifur_IRC_Cartesians_noMW_PC2.xyz

##### PC3

bifur_IRC_Cartesians_noMW_output/bifur_IRC_Cartesians_noMW_PC3.xyz

##### PCs 1-3 Combined

bifur_IRC_Cartesians_noMW_output/bifur_IRC_Cartesians_noMW_all_PCs.xyz

##### MD Trajectories A-D in PC1-3

bifur_IRC_Cartesians_noMW_output/new_data/bifur_traj_A_Cartesians_noMW_PC1.xyz

bifur_IRC_Cartesians_noMW_output/new_data/bifur_traj_A_Cartesians_noMW_PC2.xyz

bifur_IRC_Cartesians_noMW_output/new_data/bifur_traj_A_Cartesians_noMW_PC3.xyz

bifur_IRC_Cartesians_noMW_output/new_data/bifur_traj_A_Cartesians_noMW_all_PCs.xyz

bifur_IRC_Cartesians_noMW_output/new_data/bifur_traj_B_Cartesians_noMW_PC1.xyz

bifur_IRC_Cartesians_noMW_output/new_data/bifur_traj_B_Cartesians_noMW_PC2.xyz

bifur_IRC_Cartesians_noMW_output/new_data/bifur_traj_B_Cartesians_noMW_PC3.xyz

bifur_IRC_Cartesians_noMW_output/new_data/bifur_traj_B_Cartesians_noMW_all_PCs.xyz

bifur_IRC_Cartesians_noMW_output/new_data/bifur_traj_C_Cartesians_noMW_PC1.xyz

bifur_IRC_Cartesians_noMW_output/new_data/bifur_traj_C_Cartesians_noMW_PC2.xyz

bifur_IRC_Cartesians_noMW_output/new_data/bifur_traj_C_Cartesians_noMW_PC3.xyz

bifur_IRC_Cartesians_noMW_output/new_data/bifur_traj_C_Cartesians_noMW_all_PCs.xyz

bifur_IRC_Cartesians_noMW_output/new_data/bifur_traj_D_Cartesians_noMW_PC1.xyz

bifur_IRC_Cartesians_noMW_output/new_data/bifur_traj_D_Cartesians_noMW_PC2.xyz

bifur_IRC_Cartesians_noMW_output/new_data/bifur_traj_D_Cartesians_noMW_PC3.xyz

bifur_IRC_Cartesians_noMW_output/new_data/bifur_traj_D_Cartesians_noMW_all_PCs.xyz

#### Mass-Weighted

##### PC1

bifur_IRC_Cartesians_MW_output/bifur_IRC_Cartesians_MW_PC1.xyz

##### PC2

bifur_IRC_Cartesians_MW_output/bifur_IRC_Cartesians_MW_PC2.xyz

##### PC3

bifur_IRC_Cartesians_MW_output/bifur_IRC_Cartesians_MW_PC3.xyz

##### PCs 1-3 Combined

bifur_IRC_Cartesians_MW_output/bifur_IRC_Cartesians_MW_all_PCs.xyz

##### MD Trajectories A-D in PC1-3

bifur_IRC_Cartesians_MW_output/new_data/bifur_traj_A_Cartesians_MW_PC1.xyz

bifur_IRC_Cartesians_MW_output/new_data/bifur_traj_A_Cartesians_MW_PC2.xyz

bifur_IRC_Cartesians_MW_output/new_data/bifur_traj_A_Cartesians_MW_PC3.xyz

bifur_IRC_Cartesians_MW_output/new_data/bifur_traj_A_Cartesians_MW_all_PCs.xyz

bifur_IRC_Cartesians_MW_output/new_data/bifur_traj_B_Cartesians_MW_PC1.xyz

bifur_IRC_Cartesians_MW_output/new_data/bifur_traj_B_Cartesians_MW_PC2.xyz

bifur_IRC_Cartesians_MW_output/new_data/bifur_traj_B_Cartesians_MW_PC3.xyz

bifur_IRC_Cartesians_MW_output/new_data/bifur_traj_B_Cartesians_MW_all_PCs.xyz

bifur_IRC_Cartesians_MW_output/new_data/bifur_traj_C_Cartesians_MW_PC1.xyz

bifur_IRC_Cartesians_MW_output/new_data/bifur_traj_C_Cartesians_MW_PC2.xyz

bifur_IRC_Cartesians_MW_output/new_data/bifur_traj_C_Cartesians_MW_PC3.xyz

bifur_IRC_Cartesians_MW_output/new_data/bifur_traj_C_Cartesians_MW_all_PCs.xyz

bifur_IRC_Cartesians_MW_output/new_data/bifur_traj_D_Cartesians_MW_PC1.xyz

bifur_IRC_Cartesians_MW_output/new_data/bifur_traj_D_Cartesians_MW_PC2.xyz

bifur_IRC_Cartesians_MW_output/new_data/bifur_traj_D_Cartesians_MW_PC3.xyz

bifur_IRC_Cartesians_MW_output/new_data/bifur_traj_D_Cartesians_MW_all_PCs.xyz

# Plots of *PathReducer* Results

### “Distances” Input to PathReducer

#### Not Mass-Weighted

##### Proportion of Variance


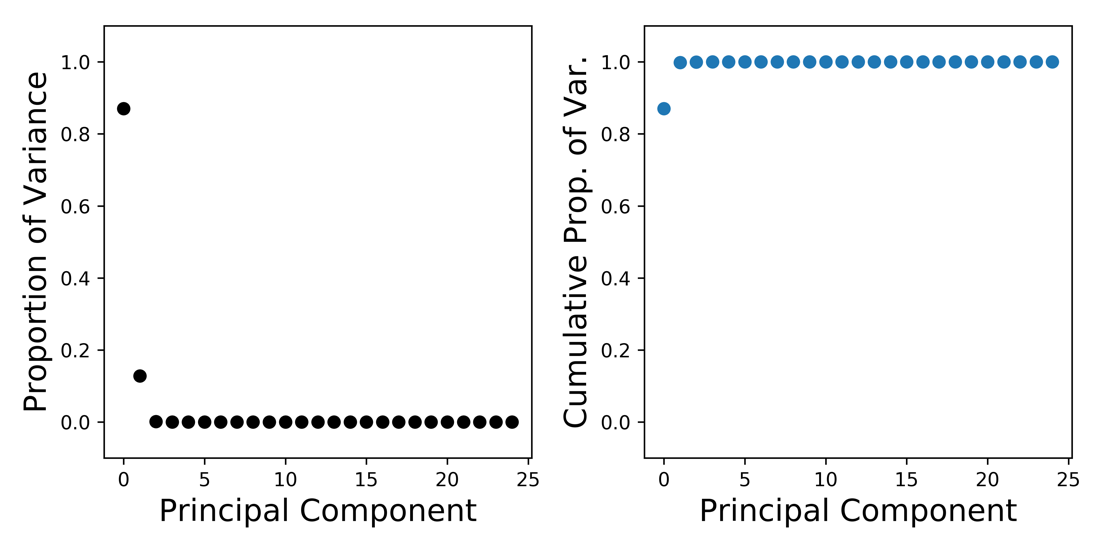


##### Paths in Top Two and Top Three PCs

#### Mass-Weighted

##### Proportion of Variance


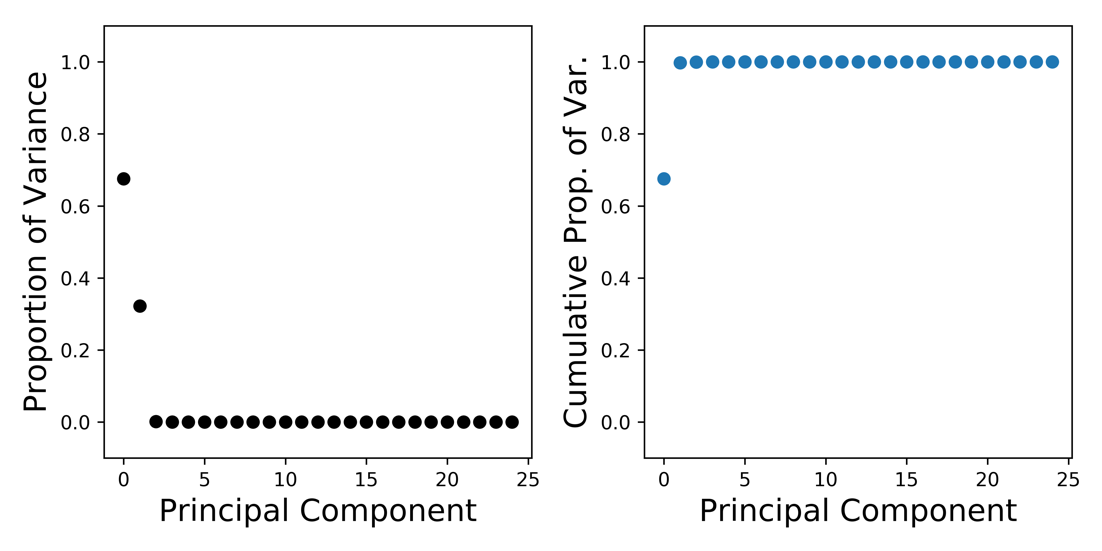


##### Paths in Top Two and Top Three PCs

### “Cartesians” Input to PathReducer

#### Not Mass-Weighted

##### Proportion of Variance


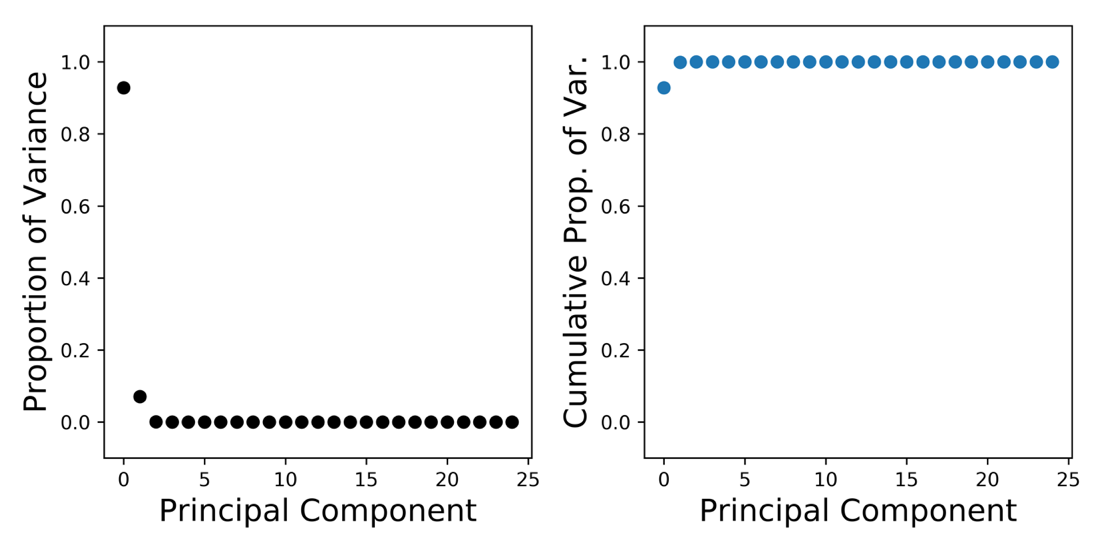


##### Paths in Top Two and Top Three PCs

#### Mass-Weighted

##### Proportion of Variance


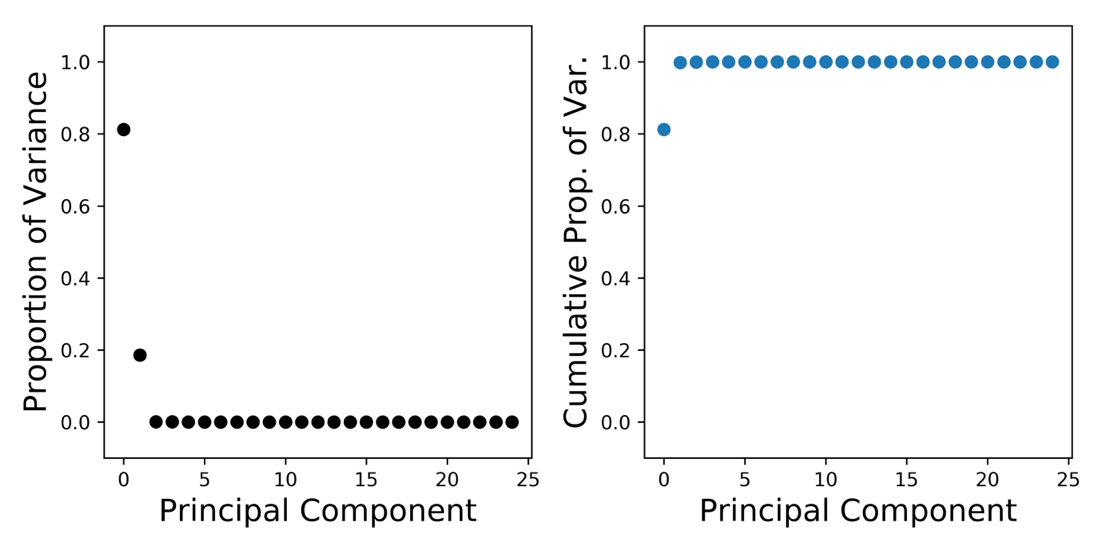


##### Paths in Top Two and Top Three PCs

## S_N_2

### “Distances” Input to PathReducer

#### Not Mass-Weighted

##### Proportion of Variance


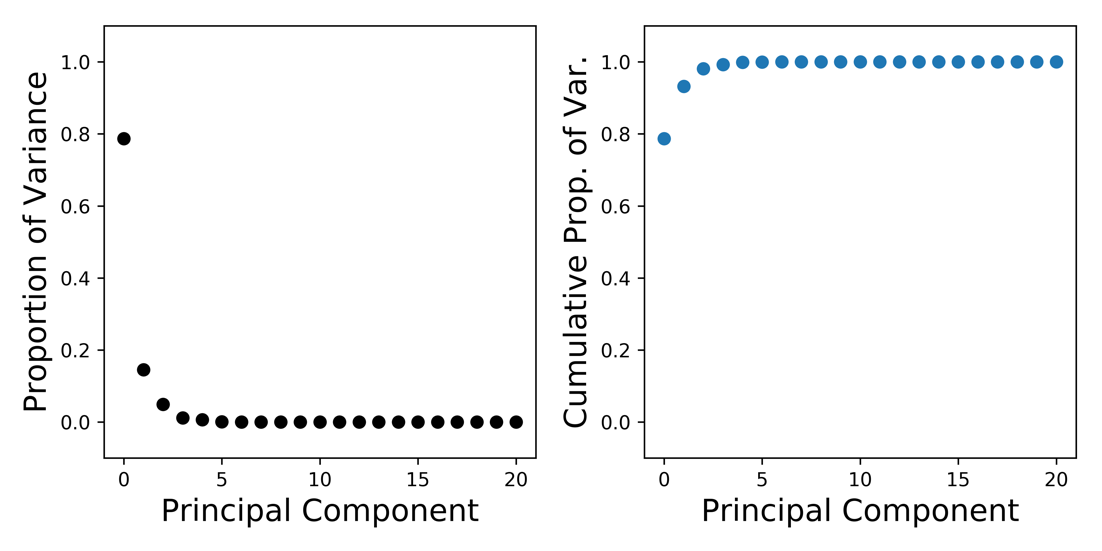


##### Paths in Top Two and Top Three PCs

##### MD Trajectory Path in Top Two and Top Three PCs

#### Mass-Weighted

##### Proportion of Variance


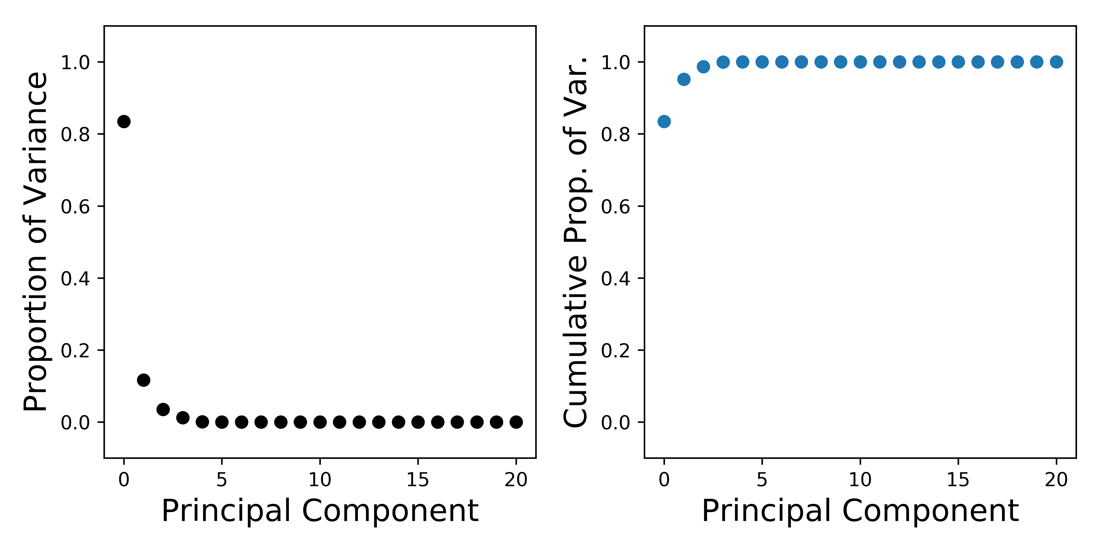


##### Paths in Top Two and Top Three PCs

##### MD Trajectory Path in Top Two and Top Three PCs

### “Cartesians” Input to PathReducer

#### Not Mass-Weighted

##### Proportion of Variance


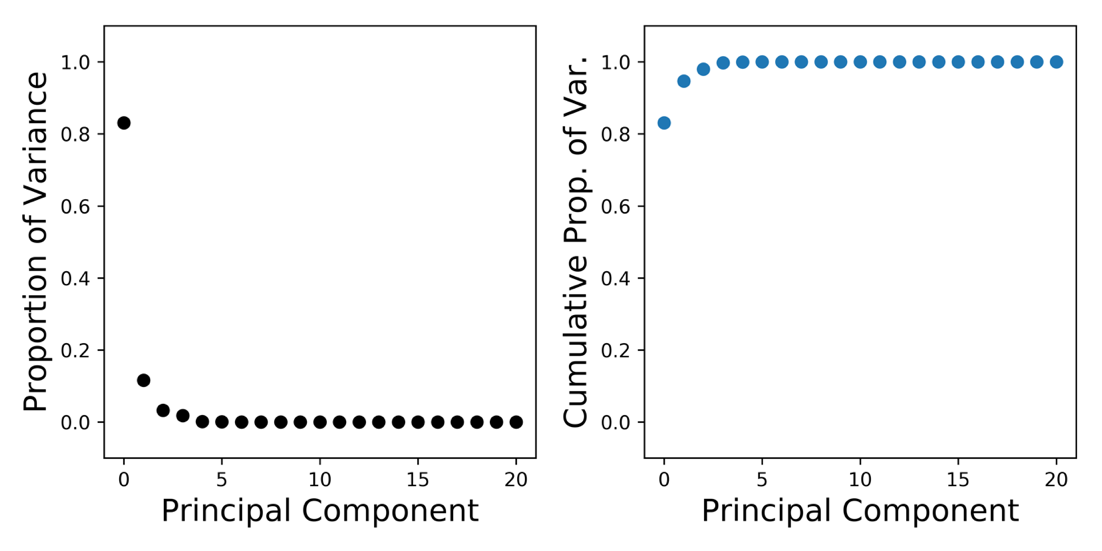


##### Paths in Top Two and Top Three PCs

##### MD Trajectory Path in Top Two and Top Three PCs

#### Mass-Weighted

##### Proportion of Variance


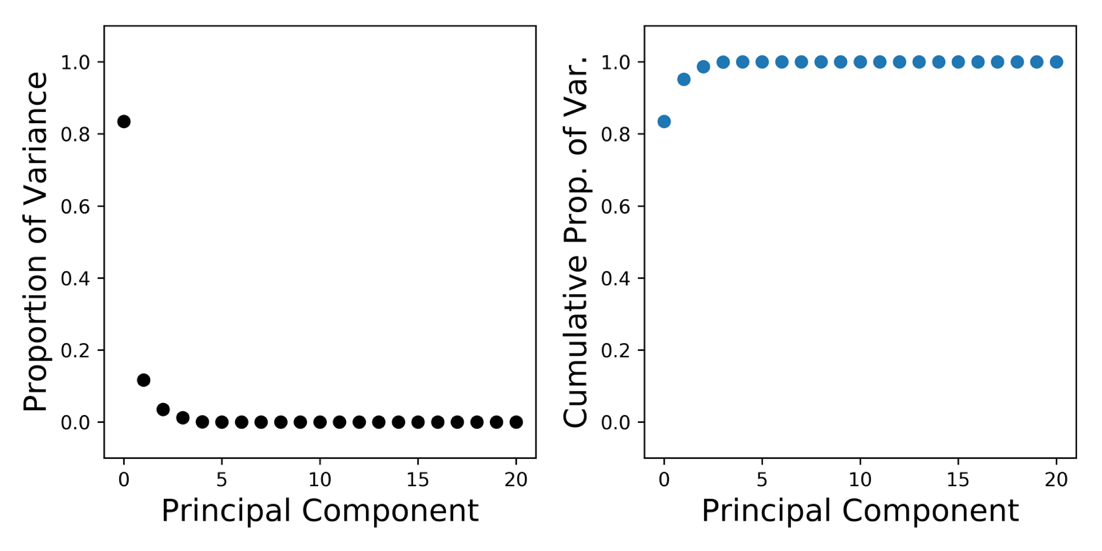


##### Paths in Top Two and Top Three PCs

##### MD Trajectory Path in Top Two and Top Three PCs

## N_2_O-Appended Acrylonitrile

### “Distances” Input to PathReducer

#### Not Mass-Weighted

##### Proportion of Variance


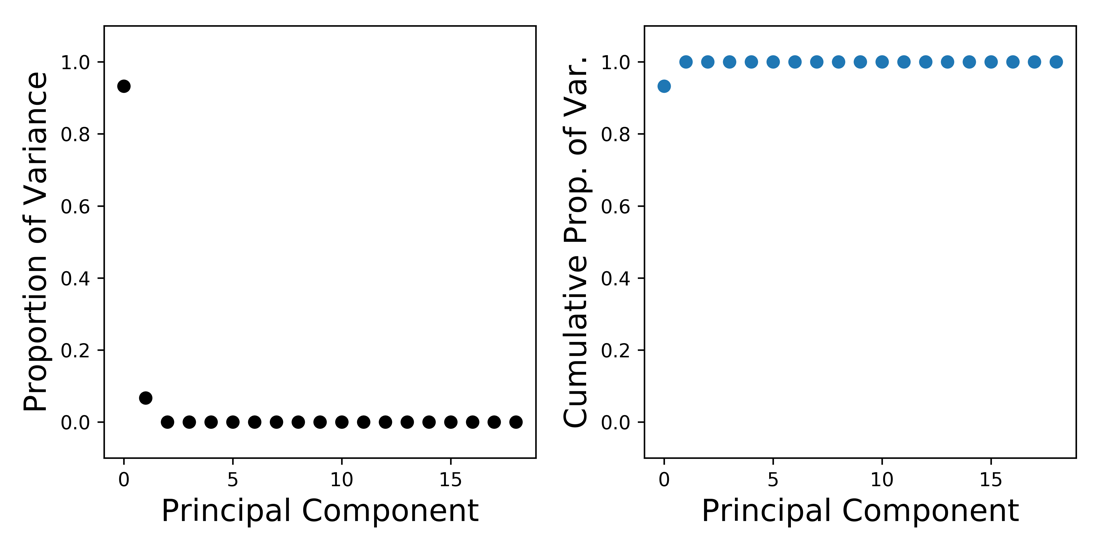


##### Paths in Top Two and Top Three PCs

#### Mass-Weighted

##### Proportion of Variance


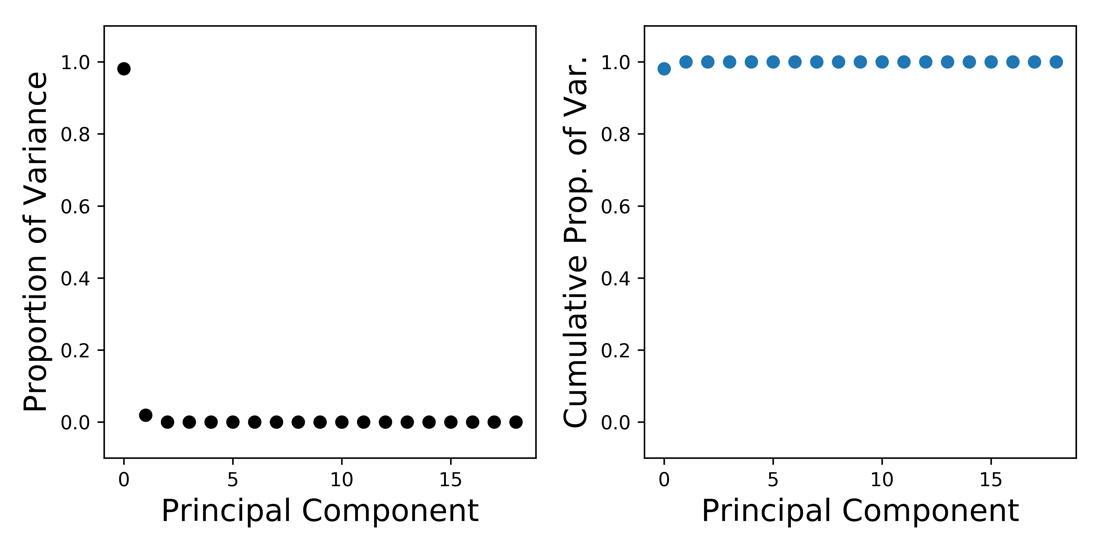


##### Paths in Top Two and Top Three PCs

### “Cartesians” Input to PathReducer

#### Not Mass-Weighted

##### Proportion of Variance


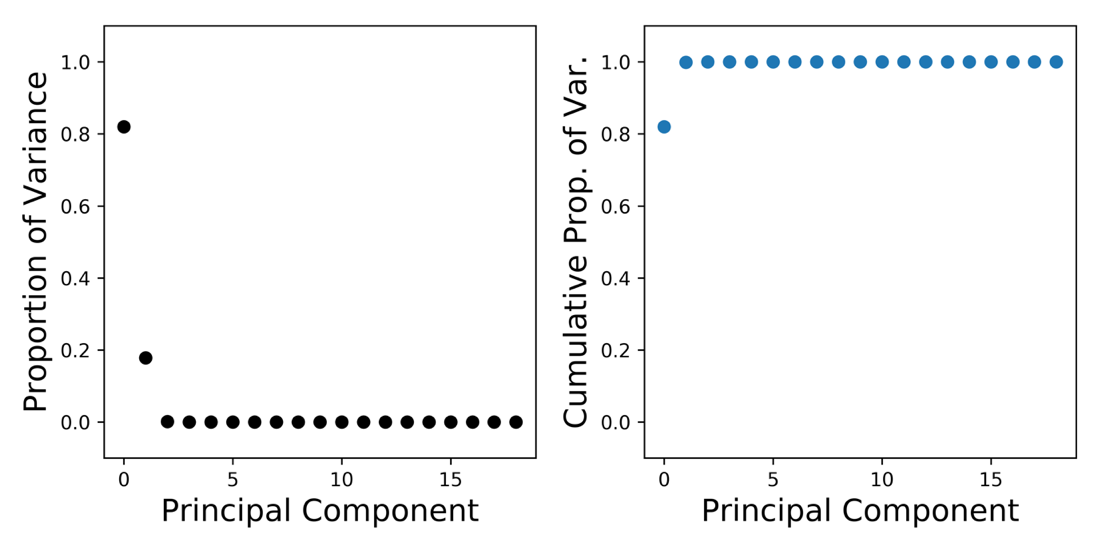


##### Paths in Top Two and Top Three PCs

#### Mass-Weighted

##### Proportion of Variance


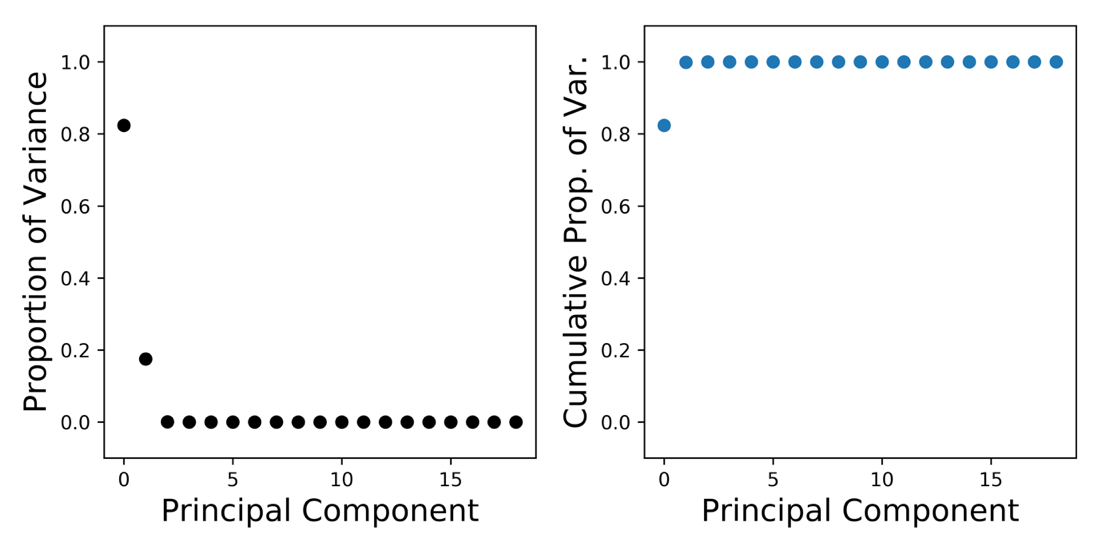


##### Paths in Top Two and Top Three PCs

## Cyclopropylidene Bifurcation

### “Distances” Input to PathReducer

#### Not Mass-Weighted

##### Proportion of Variance


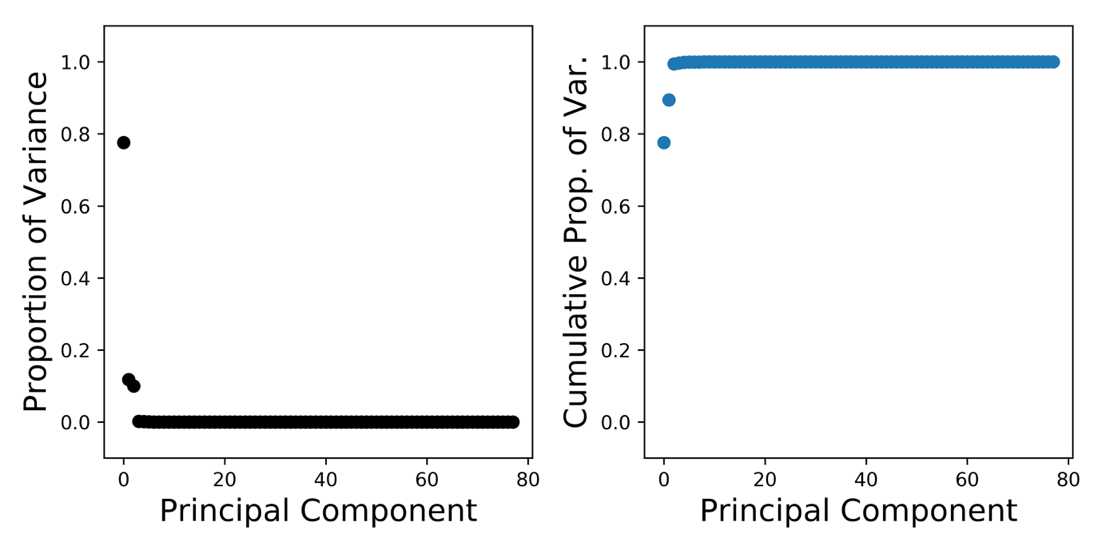


##### Paths in Top Two and Top Three PCs

##### MD Trajectory A Path in Top Two and Top Three PCs

##### MD Trajectory B Path in Top Two and Top Three PCs

##### MD Trajectory C Path in Top Two and Top Three PCs

##### MD Trajectory D Path in Top Two and Top Three PCs

#### Mass-Weighted

##### Proportion of Variance


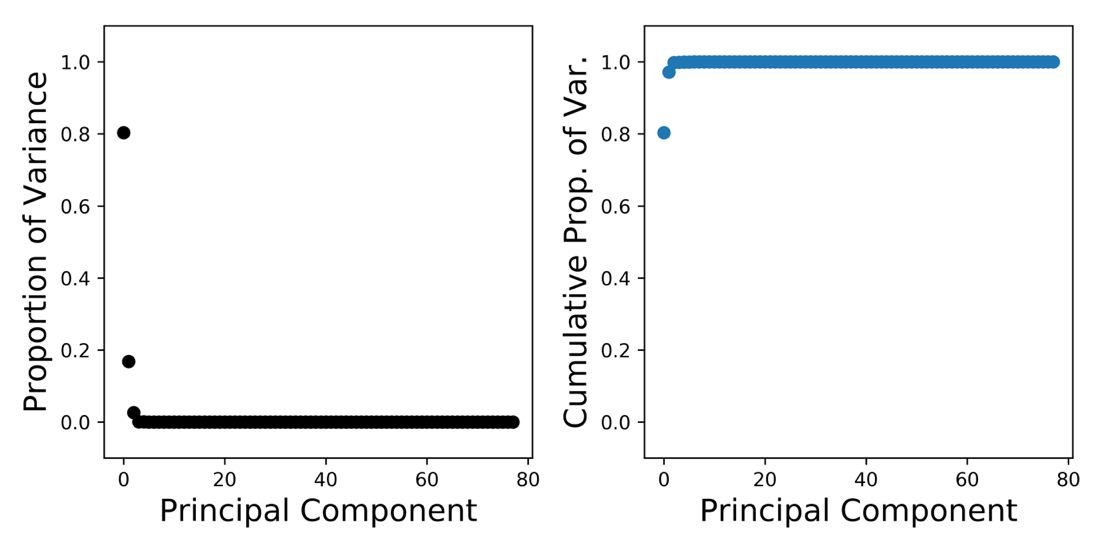


##### Paths in Top Two and Top Three PCs

##### MD Trajectory A Path in Top Two and Top Three PCs

##### MD Trajectory B Path in Top Two and Top Three PCs

##### MD Trajectory C Path in Top Two and Top Three PCs

##### MD Trajectory D Path in Top Two and Top Three PCs

### “Cartesians” Input to PathReducer

#### Not Mass-Weighted

##### Proportion of Variance


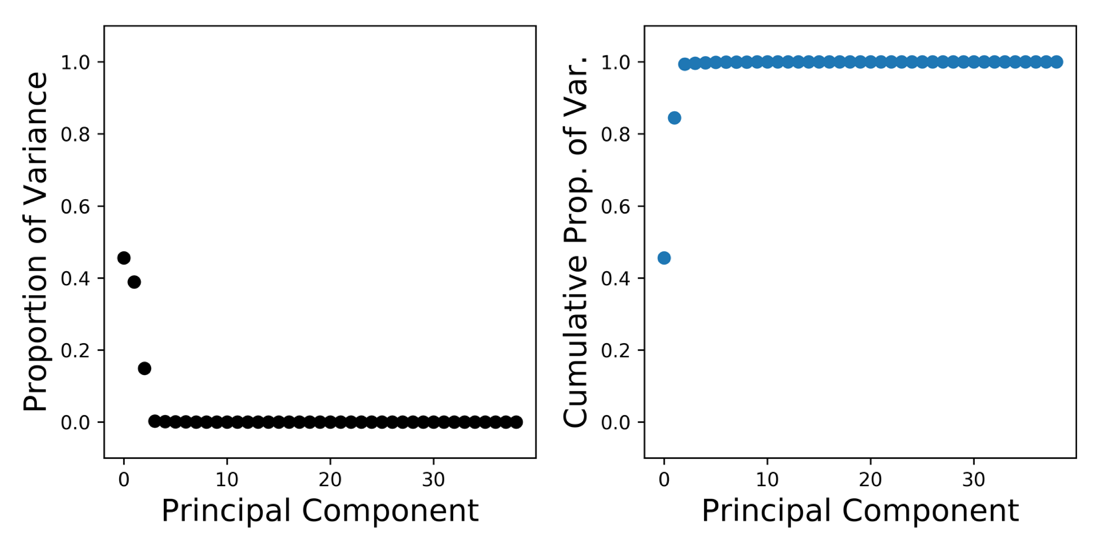


##### Paths in Top Two and Top Three PCs

##### MD Trajectory A Path in Top Two and Top Three PCs

##### MD Trajectory B Path in Top Two and Top Three PCs

##### MD Trajectory C Path in Top Two and Top Three PCs

##### MD Trajectory D Path in Top Two and Top Three PC

#### Mass-Weighted

##### Proportion of Variance


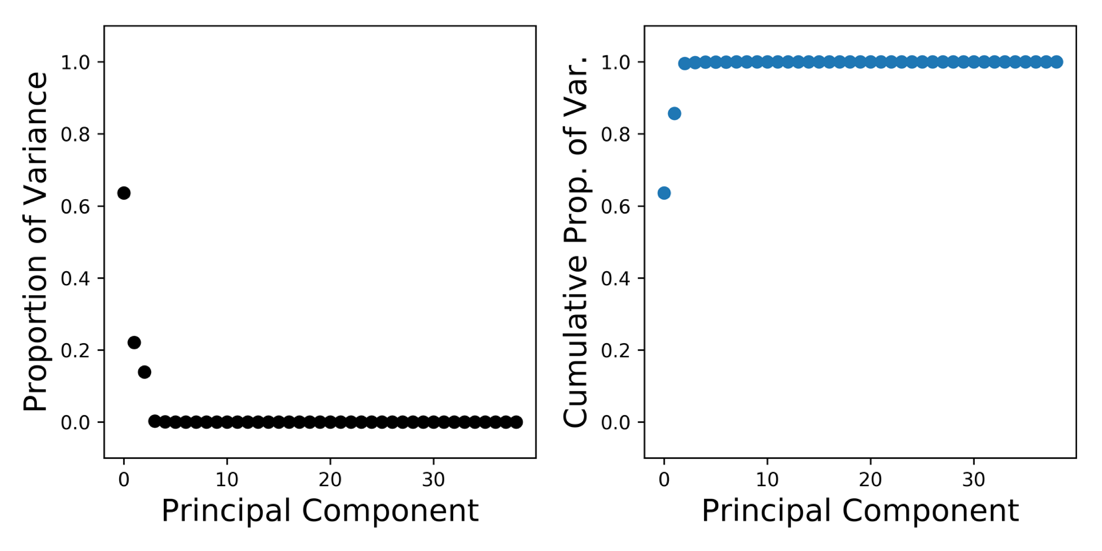


##### Paths in Top Two and Top Three PCs

##### MD Trajectory A Path in Top Two and Top Three PCs

##### MD Trajectory B Path in Top Two and Top Three PCs

##### MD Trajectory C Path in Top Two and Top Three PCs

##### MD Trajectory D Path in Top Two and Top Three PCs

# Gaussian BOMD input

The following lines show the input to a Gaussian Born-Oppenheimer Molecular Dynamics (BOMD) simulation for the S_N_2 example system, initiated from the transition state structure. This simulation was run with a step size of 1 fs, a rotational temperature of 100K, and 1kcal/mol in the imaginary vibrational mode. This particular input propagates the trajectory forward in time, which in this case corresponds to moving in the direction of the product. To propagate a trajectory backward in time (in this case, to get the half of the trajectory that goes from transition state structure to reactant), the IOp keyword needs to be set to “IOp(1/105=20)”.

%chk=SN2_dyn_BOMD_1kcal.chk

%mem=1GB

%nprocs=40

# mp2/6-31+g(d) BOMD(stepsize=1000, maxpoints=500, NSample=1, phase=(1,3), rtemp=100) IOp(1/105=10)

TS hydroxide SN2 on fluoromethane

-1 1

C -0.02848501 -0.01625247 0.00002685

O 1.98498699 0.09336353 -0.00001115

F -1.78367901 -0.02321847 -0.00001215

H 0.03028999 -0.54707947 -0.93321915

H 0.03024999 -0.54698247 0.93331885

H 0.04965399 1.05619853 -0.00002115

H 2.34973299 -0.81267947 0.00000785

1

1 1 2 1 1 1 1

1 1.0
